# The Polynomial Progression Subtype Inference Algorithm

**DOI:** 10.21203/rs.3.rs-7199106/v1

**Published:** 2025-10-07

**Authors:** August F. Van Hout, Stiven Roytman, Giulia Carli, Travis P. Wigstrom, Prabesh Kanel, Nicolaas I. Bohnen

**Affiliations:** 1 Department of Radiology, University of Michigan, Ann Arbor, MI, USA.; 2 Functional Neuroimaging, Cognitive, and Mobility Laboratory, Department of Radiology, University of Michigan, Ann Arbor, MI, USA.; 3 Morris K. Udall Center of Excellence for Parkinson’s Disease Research, University of Michigan, Ann Arbor, MI 48109, USA; 4 Department of Neurology, University of Michigan, Ann Arbor, MI, USA.; 5 Neurology Service and GRECC, VA Ann Arbor Healthcare System, Ann Arbor, MI 48105, USA

## Abstract

Longitudinal assessments are currently the gold standard for modeling progression of diseases, but they delay prognosis and increase burden on patients and healthcare systems. Cross-sectional inference offers a valuable alternative, enabling earlier patients’ stratification and broader accessibility. Initial success in this direction has been found with the SuStaIn algorithm (Young et al. 2018) but computational and conceptual shortcomings hamper its usefulness. Here we introduce a more effective algorithm, PPSI, which is orders of magnitude faster, easier to interpret, equally or more accurate, applicable to more complex bidirectional phenomena, and can be fitted with many more variables at once. We demonstrate PPSI’s utility using longitudinal prediction in Alzheimer’s disease (ADNI database), clinical subtype recovery in breast cancer (TCGA-BRCA), and measurement of robustness under simulated conditions. To promote broad usability, we provide an extensive plotting suite for model exploration and diagnostics, and a graphical user interface that allows non-programmers to use the tool. While PPSI and SuStaIn are both able to derive useful subtypes from data, PPSI dramatically improves computational efficiency, enabling the inclusion of thousands of features and reducing runtimes from hours to seconds. PPSI is a performant interactive tool which makes disease progression modeling accessible to any subject matter expert.

## Introduction

### Progressions

In studying progressive phenomena such as growth, decay, or disease, longitudinal data are essential for understanding temporal dynamics. However, in many real-world settings, repeated measurements are costly, time-consuming, or impractical. This creates a critical need for models that can infer temporal progression and stratify individuals based on a single cross-sectional observation. Such tools are especially valuable for prognosis and treatment planning, enabling early stratification without requiring multiple follow-up assessments. While longitudinal data remain fundamental for validating these models, the ability to make reliable inferences from cross-sectional data holds promise for reducing diagnostic delays and patient burden. This work provides such a methodology, deriving subtypes from a dataset of observations and representing each subtype’s progression through time using polynomial equations. We model change in multiple variables over time and simultaneously separate the dataset into groups which progress differently from one another.

The algorithm is intended for use on cross-sectional datasets where each row (or record) is an observation and each column (or feature, variable) is a measurement. In such datasets, all feature measurements are taken at approximately the same time for each observation. Multiple observations of the same subject over time are not required, though highly useful in validating inferred progression models, as we will demonstrate.

This logical challenge of establishing groups of observations within cross sectional datasets has long been addressed using traditional clustering methods such as hierarchical clustering, k-means clustering, and gaussian mixture models (Cacciapaglia et al. 2024; Levin et al. 2021). While these methods can yield meaningful clusters, they are less suited to model long continuous trajectories (progressions). Further, some phenomena may be better described by a set of multiple independent trajectories, entangling the contributions of progression time and subtype to the resulting clusters. There exists a need to arrange cross sectional datasets of progressive phenomena into the most likely order (staging) while also separating them to allow for multiple divergent progressions within the same dataset (subtyping).

#### Previous Algorithms

Modeling longitudinal progression subtypes using cross-sectional data is an active area of study. Early methods included temporal bootstrapping with hidden Markov models to connect healthy and disease states (Li, Swift, and Tucker 2013) which found some successes but were not widely adopted. Subtype and Stage Inference (SuStaIn) is among the most recent and successful algorithms addressing this problem (Young et al. 2018) and serves as a starting point for this work.

SuStaIn captures progression by estimating the “disease time points” at which different features cross a set of normative thresholds. Subtypes are obtained by splitting the dataset repeatedly and estimating the progression-related disease time points independently for each split until the likelihood of the data given the model is maximized. As an accessible solution to the problem of modelling longitudinal progression subtypes from cross-sectional data, SuStaIn has been widely applied in the field of disease progression modeling, where it has been used to shed greater light on the progression of Alzheimer’s disease (AD) (Aksman et al. 2023; Young et al. 2018), fronto-temporal dementia (Young et al. 2018), multiple sclerosis (Eshaghi et al. 2021), and progressive supranuclear palsy (H. Wang et al. 2024).

Despite its popularity and effectiveness, SuStaIn carries some limitations scarcely discussed in prior literature. Namely, there are restrictions to the shape of progressions which can be modeled, high computational complexity which limit the number of features used in training, limitations to usability and interpretability, and that SuStaIn’s efficacy is less often formally measured against already-known progressive disease ground-truths.

First, SuStaIn cannot model features which change direction and travel back across normalcy. Because SuStaIn estimates the progression times at which each feature passes through a set of normative thresholds, it requires that the thresholds only be crossed once, and in only one direction away from the progression’s origin. In the domain of neurodegenerative disease, such non-monotonic biomarker progressions exist. For example, compensatory increases in cerebral glucose metabolism linked to pro-inflammatory microglial states have been previously reported in AD (Biel et al. 2023; Choi et al. 2021), preceding better recognized cerebral hypometabolism of AD (Raut et al. 2023). In Parkinson disease (PD), upregulation of cholinergic nerves terminal activity and density are linked with preserved cognition in recently diagnosed patients (Legault-Denis et al. 2021; van der Zee et al. 2022; Legault-Denis et al. 2024), also as an antecedent of widespread loss of cerebral cholinergic nerve terminals observed in advanced disease stages (Bohnen et al. 2022).I

Second, SuStaIn is computationally intensive. SuStaIn utilizes Markov-chain Monte Carlo (MCMC) to estimate the times at which biomarkers will cross pre-specified thresholds away from normalcy, performing this optimization process on many random splits of the dataset to uncover subtypes (Young et al. 2018). MCMC can be computationally inefficient, and SuStaIn scales poorly with the number of features in the dataset; the authors found a practical limit, using normal 4–6 core computers, of around 30. Application of SuStaIn to feature-rich datasets thus requires manual feature reduction, precluding many applications and hampering exploratory analyses. This issue is most apparent when cross-validating SuStaIn models, as recommended by the author (Young et al. 2018), to determine the optimal number of subtypes. While a more efficient permutation of SuStaIn, s-SuStaIn (Tandon, Lah, and Mitchell 2024), has recently been published, fitting times in feature-rich datasets (up to 200 features tested) may reportedly take up to 10 minutes on a high performance 48-core computer.

Third, SuStaIn is conceptually complex, and the threshold-crossing timepoints arrived at through SuStaIn’s optimization process are not very intuitive to interpret. Further, that set of feature thresholds for which SuStaIn finds optimal crossing timepoints must be specified a-priori by the user, with defaults at 1, 2, and 3 standard deviations away from normative values. These values are not always the best fit, and little guidance for choosing different thresholds is provided. A clear improvement for subtyping and staging algorithms would be a parametric estimation of continuous feature trajectories in a solution space conceptually closer to a smooth progression.

Finally, SuStaIn’s efficacy yet lacks a wealth of formal quantifications. There is a need for more publications verifying that longitudinally observed real-world subjects modeled with SuStaIn at baseline do not, at follow up, a) travel backwards in their progression, and b) change subtype, as few such papers have been published (Estarellas et al. 2024). It is a healthy measure for algorithms aiming to infer longitudinal progression from cross-sectional data to formally demonstrate that the inferred progressions are consistent with longitudinal ground truths. Also, when publishing an algorithm with subtyping functionality, a demonstration that the method captures already-understood subtypes in a thoroughly studied field is of interest. We perform all these validations in this initial work.

We shall now introduce and validate a subtyping and staging algorithm which is capable of modeling bidirectional changes through time, utilizes highly efficient optimization methods, and which is meaningfully simpler in design.

#### The PPSI Algorithm

Polynomial progression subtype inference (PPSI) falls under a class of “phenomenological subtyping” progression models. It does not model the underlying processes of disease progression, but rather learns a set of feature trajectories along a data-driven time axis that offers best fit to the data (Young et al. 2024). Very simply, PPSI models each biomarker as a polynomial equation over time and can produce models with multiple subtypes progressing differently within the same dataset. The polynomial equation of each feature is modeled with a fixed intercept term set at 0 (the mean of normative values) and evaluated at a discrete number of evenly spaced time-points (stages), across a time domain between 0 (beginning of progression) and 1 (end of progression).

Negative Log Likelihood is used as our loss function. At each stage, one can evaluate all feature polynomials to produce a point in multidimensional space. If we consider this point as the centroid of a multidimensional cluster (stage-cluster), we can evaluate the likelihood of an observation belonging to that stage. During optimization, a PPSI model iteratively updates the polynomial coefficients to maximize the likelihood of each observation evaluated at its most likely stage-cluster.

To capture multiple potentially heterogeneous progression subtypes within the same dataset, another set of independent polynomial equations may be instantiated and fitted to the data in parallel, making available an additional set of stage-clusters along an independent progression to which observations may be assigned. The subtype to which the maximal likelihood stage-cluster of a given observation belongs is the subtype assigned to that observation. A mathematical explication is provided in the Methods section “Algorithm Mathematics”, and a plain-english explanation of the algorithm is given in Supplementary Material 1.

#### Advantages of PPSI

Modeling feature progressions as continuous polynomial equations overcomes multiple limitations of SuStaIn. First, it allows for modeling non-monotonic feature progressions by making use of mutual information between features. As long as at least one feature has a reliable monotonic progression, it should be possible to disambiguate the early and late phases of the others.

Second, it can be made orders of magnitude faster. Because the trainable model weights are polynomial equation coefficients, it is possible to train the model with the much more efficient gradient descent optimization strategy. Fast and reliable convergence is ensured with the ADAM optimizer (Kingma and Ba 2014) and an annealing-based learning-rate scheduler, similar to the approaches commonly employed to train deep-learning models (Nakamura et al. 2021). Our implementation of the algorithm is also written in the highly-performant Julia programming language, and may utilize multi-core modern processors. On a common computer, interactive exploratory analysis and model cross-validation are momentary affairs, and it is possible for the algorithm to train on thousands of features at a time.

We leverage the computational efficiency of the training process and the intuitive nature of PPSI model results to introduce a novel paradigm of progression modeling methodology which encourages the user to actively explore and critically scrutinize iteratively trained PPSI models using visualizations, robust performance metrics, and cross-validation. Our open-source Julia software library, which we make publicly available, already implements a diverse toolbox of these methods.

We also implement a built-in module for generating datasets based on simulated ground truth polynomials. Users can simulate various adverse factors that might compromise their ability to accurately stage and subtype their data (high feature noise, class inequality between subtypes, poor feature usefulness, low sample size or feature count, uneven sampling across the progression, and more).

Finally, we release PPSI with a simple interactive GUI for those who are experts in their scientific domain rather than experts at coding in Julia. In the PPSI repository can be found an interactive Pluto notebook wherein users can upload datasets, z-score them, iteratively fit models, and evaluate those models using all plots from PPSI’s subject matter expert plotting suite. This notebook has been packaged into an executable file with which users are encouraged to explore PPSI.

#### Application

To evaluate our novel methodology for inferring longitudinal progressions from cross-sectional data, we first applied it to the Alzheimer’s Disease Neuroimaging Initiative (ADNI) dataset (Petersen et al. 2010) and to a breast cancer protein expression dataset from the National Cancer Institute’s Genomic Data Commons (GDC) Data Portal (Heath, A.P., Ferretti, V., Agrawal, S. et al. 2021). We then applied the algorithm to many simulated ground-truth datasets in two simulation studies.

We use the ADNI dataset to demonstrate an application of PPSI to a multi-biomarker cross-sectional training dataset. The resulting model is then validated on a hold-out set of longitudinal observations to demonstrate that obtained subtypes are longitudinally consistent and that the within-subject stage progression from baseline to follow-up proceeds predominantly in the expected direction. We perform a head-to-head comparison between PPSI and SuStaIn based on a manually reduced feature set which SuStaIn can process. We also perform tests of cross-sectional and longitudinal predictive validity by showing that the subtype and stage assignments derived from the resulting PPSI model are able to predict follow-up dementia status on the hold-out validation set better than a null model containing only baseline dementia status and number of days between baseline and follow-up visits.

We then apply PPSI to a dataset of breast cancer tumor protein expressions with two goals: first, to demonstrate a single iteration of our recommended modeling process, and second, to determine if PPSI can detect meaningful subtypes in a field where subtyping work has previously been done. We create models using all protein variables, perform a post-hoc feature-importance based variable reduction, and then repeat the modeling process on this reduced feature set before using the final selected model for clinical validation analyses. We assess the subtyping capabilities of the model by comparing the obtained data-driven subtypes against ground truth breast cancer subtypes.

Finally, we perform two types of simulation studies. The aim of the first is to explore the algorithm and guide its design, and the aim of the second is to test its effectiveness in progressively adverse conditions. In both studies, subtyping performance is measured using the Adjusted Rand Index (ARI) of ground-truth versus PPSI assigned subtypes, and staging performance is measured using the Pearson’s R correlation coefficient of ground-truth stages versus PPSI-assigned stages.

All computations were performed using everyday hardware which real-world practitioners are likely to be using, including a 4-core mac and a 6-core PC.

#### Alzheimer’s Study

##### Dataset description

Details on data curation and image preprocessing for the ADNI dataset are provided in Supplementary Material 2. The training dataset had a total of 89 features, consisting of 88 bilaterally averaged regional PET uptake values (44 glucose PET, 44 amyloid PET), and ABETA42 CSF biomarker (P-TAU CSF biomarker was held out for external validation). Each observation was a set of measurements made on an AD patient (see methods section for definition), with a total of 249 cross-sectional observations in the training set. A total of 55 additional patients with baseline and follow-up observations were held out from PPSI model training to be used for longitudinal and external validation along with out-of-sample evaluation of model performance.

##### Model building and evaluation

Exploratory analyses indicated clearest support for a 2 subtype, 2nd degree polynomial model (see Extended Data section 1). The subtypes captured by the 2-subtype model appear to reflect a differential progression of cerebral amyloid and glucose uptake associated with AD progression. Extended Data section 2 contains these visualizations. The first subtype appears to manifest a pattern of cerebral glucose hypermetabolism earlier in the progression, followed by rapidly emerging hypometabolism, in conjunction with a more pronounced increase in cerebral amyloid deposition (hypermetabolic subtype). Second subtype appears to manifest only cerebral glucose hypometabolism in conjunction with a more gradual cerebral amyloid buildup (hypometabolic subtype). CSF ABETA42 progression does not appear strongly differential between the two subtypes.

##### Longitudinal Validation

We performed a longitudinal validation of the 2-subtype model based on a sample of 55 AD patients with baseline and follow-up observations that were held out from the training set. Detailed methods for longitudinal validation on ADNI dataset are provided in the “ADNI Longitudinal Validation” section of the methods. On average, the time difference between baseline and follow-up observations was 845.7 days (CI95=[760.7, 930.7] days). Three longitudinal validity measures were computed to characterize the 2-subtype model: subtype consistency (proportion of observations for which the baseline and follow-up subtype match), stage monotony (proportion of observations for which the follow-up stage is greater than or equal to baseline stage), and stage progression (proportion of observations for which the follow-up stage is greater than the baseline stage).

For subtype consistency, a metric value of 0.816 (CI95=[0.794, 0.839]) was observed for the 2-subtype model on the longitudinal validation set, which was greater (p < 0.001) than the value that is expected by chance alone (0.504, CI95=[0.5, 0.508]). For stage monotony, a metric value of 0.731 (CI95=[0.719, 0.743]) was observed, which was greater (p < 0.001) than the value that is expected by chance alone (0.591, CI95=[0.588, 0.593]). For stage progression, a metric value 0.6 (CI95=[0.59, 0.61]) was observed, which trended towards, but failed to reach, statistical significance (p = 0.063) towards being greater than what would be expected based on chance alone (0.54, CI95=[0.537, 0.542]). In conclusion, these findings demonstrate that PPSI yields subtypes which are longitudinally stable and generally proceed in the expected direction. Failure to observe statistically significant stage progression at a level greater than chance might relate to the distribution of stages at baseline, which was not controlled for in the present analysis.

Lastly, a head-to-head comparison of longitudinal validation metrics was performed between PPSI and SuStaIn on a reduced dataset of 15 features, including ABETA42, TAU, and P-TAU CSF biomarkers, along with regionally averaged amyloid and glucose PET values. Higher level regions were defined based on a-priori anatomical boundaries (see list of regions and corresponding definitions listed in “ADNI Longitudinal Validation” methods section). In cases of both SuStaIn and PPSI, a 2 subtype model was fitted. A subtype consistency of 0.8 was observed for PPSI and of 0.818 for SuStaIn. Stage monotony of 0.836 was observed for PPSI, and 0.8 for SuStaIn. Stage progression of 0.6 was observed for PPSI and of 0.586 for SuStaIn. In conclusion, subtypes and stages obtained both with PPSI and SuStaIn appear to have acceptable and effectively equivalent longitudinal validation metrics.

##### Clinical Predictive Validity

As one of the external validity tests for PPSI on the ADNI dataset, we attempted to predict follow-up dementia status in the held-out validation set of 55 Alzheimer’s patients with longitudinal data based on subtype and stage inferred at baseline. Four out of the 55 patients had dementia status at baseline, while at follow-up, 16 out of 55 patients had dementia status. Detailed methods for clinical predictive validity logistic regression modelling are presented in the “Clinical Validation” section of the methods.

A subtype by stage interaction model offered better predictive performance and fit to the data than the model with baseline dementia status alone. The only statistically significant regressor in the logistic regression model was the marginal effect of stage (β=0.72, CI95=[0.12, 1.32], p=0.018), which suggested that for patients in the hypermetabolic subtype, with mild cognitive impairment but no dementia at baseline, every unit increase in baseline PPSI stage above the average stage of 11.2 corresponded to a 2.05 times increase in probability of having dementia at a follow-up (on average ~2 year later). At the mean stage of 11.2, the hypometabolic subtype patients trended towards greater likelihood of dementia at ~2 year follow-up than hypermetabolic subtype patients (β=2.27, CI95=[−0.83, 5.38], p=0.15), and also towards lower increase in follow-up probability of dementia as a function of stage (β=−0.53, CI95=[−1.18, 0.13], p=0.12), though the regression coefficients corresponding to these marginal and interaction effects fell short of statistical significance.

##### Breast Cancer Study

We applied PPSI to the NIH Genomic Data Common breast cancer data set. This was chosen due to breast cancer being clearly delineated by receptor subtypes in a clinical setting; notably, the presence of the Estrogen Receptor (ER), Progesterone Receptor (PR) positive, human epidermal growth factor receptor 2 (HER2) positive. The presence or absence of these receptors are used to create the clinical subtypes of Triple Negative Breast Cancer (ER, PR, and HER2 negative), HER2+ breast cancer, and Luminal A breast cancer (ER and PR positive, HER2 negative). Of note, though these are some of the most common breast cancer subtypes, this list is not exhaustive.

##### Model Building

Specifics about the dataset and data filtration, z-scoring, model fitting, and model selection are detailed in Supplementary Material section 4. Notably, 561 tumor-tissue sample records were analyzed to assess the progressions of 482 measured proteins. The protein values were z-scored using 22 non-tumorous tissue samples from within the patient population, and a modeling sweep was performed as described in Supplementary Materials. Then, 37 of the most impactful proteins were selected via PPSI’s data-driven variable reduction dropout report detailed in Supplementary Material 5. Finally, another sweep was performed, resulting in the selection of a 3 subtype, 2nd order polynomial progression model. Modeling plots can be found in Extended Data Section 3.

##### Subtype characteristics

What resulted was a three-subtype model fitted by second order polynomial functions describing those 37 selected proteins. Subtypes 1, 2, and 3 included 160, 272, and 109 individuals, respectively. We shall analyze proteins with pronounced differences and then verify that the subtypes match a standard cancer subtyping paradigm.

AR, ERK5-BMK1, CHD1L, cGAS, Merit40, and UQCR2 proteins had the most visually pronounced difference in progression by subtype. All of these proteins have biological roles concerning breast cancer. The Androgen receptor promotes cell proliferation and interacts with the estrogen receptor affecting treatment in ER and PR positive breast cancer (Klarica Gembić et al. 2024; Xu et al. 2024; Toàn 2024). BMK1 is a protein kinase involved in cell proliferation which is correlated with treatment resistance (Yang and Lee 2011), CHD1L is involved in regulation of gene expression and is associated with aggressive tumor subtypes (Corso et al. 2020; Yadav et al. 2021), cGAS is involved in the body’s immune response (Chen et al. 2024), Merit40 regulates cell survival and inflammation (Taouis et al. 2023; Brown et al. 2015), and UQCRC2 plays a role in metabolism and is associated with more aggressive tumors (Han et al. 2019; D.-W. Wang et al. 2020).

A linear model was made to quantify the interaction of stage and subtype versus protein value. The models were made with an intercept at zero (origin) and contrast against most numerous subtype 2. The results are shown below in Table 2. Supplemental Material Section 6 containing a table for all of the proteins’ individual slopes.

##### Validation

The algorithm was then validated by comparing receptor statuses of the three groups using categorical receptor status variables for ER, PR, and HER2 already present in the dataset. The vast majority of receptor values were either “positive” or “negative,” especially for Progesterone and Estrogen receptors. In the case of HER2, 94 records were also “equivocal.” Less than 6 records were “indeterminate.”

First, the very absence of categorical data was statistically significantly different between subtypes. A Chi Square test (χ2(2)=20.40, n=561, p<0.001) on a contingency table of missingness across subtypes revealed that subtype 3 was much more likely to be missing at least one categorical variable (std. resid=−4.37) and subtype 1 was more likely to have complete data (std. resid=3.21). However, the aim of this analysis is to verify that clinically meaningful subtypes can be derived using PPSI, so the remainder of this analysis was performed after dropping all records with categorical receptor values that are missing, indeterminate, or equivocal. 456 records remained.

A frequency table was generated for all combinations of receptor values for all three subtypes. A full tabulation of the results can be found in Extended Data section 4. In brief, we found prevalence of Triple-Negative breast cancer, determined as having negative receptor values for ER, PR, and Her2, and also Luminal A cancer, characterized by positive ER and PR, with negative HER2. A chi-square test (χ2(2)=105.83, p<1e-22) was used to determine disproportionate presence of triple negative records across subtypes, revealing that subtype 1 (std. resid=10.14) was far more likely to be triple negative while subtypes 2 (−8.34) and 3 (−1.19) were less likely. Another chi-square test (χ2(2)=36.96, p<1e-8) found that subtype 1 (std. resid=−5.9) was much less likely to be Luminal A, and that subtype 2 (5.2) was much more likely, with subtype 3 (0.25) near expected. This would indicate that the progression of cancer protein expression has been stratified into these respective subtypes by PPSI.

##### Comparison to SuStaIn

To compare PPSI to SuStaIn, a SuStaIn model was made using the recommended workflow. Their solutions were compared to one another and then compared to ground-truth receptor subtypes. Because SuStaIn cannot be practically trained with standard hardware on more than thirty features or so, 10 proteins were selected based on their merit in describing breast cancer progression and subtyping. The proteins, namely P53, BCL2, AKT, CYCLIND1, EGFR, BRCA2, MTOR, PDL1, FOXO3A, and PARPCLEAVED, were used to fit the SuStaIn model. Fitting was performed up to four subtypes, which took 4.56 hours. A three-subtype model was selected because the fourth additional subtype yielded much lesser gain in likelihood, as recommended by SuStaIn’s authors, not out of analytical convenience.

This SuStaIn model was compared both to a PPSI model with these 10 features and to the full 37 feature PPSI model. All models were trained on the full dataset of 561 records. Notably, the 10-feature PPSI model, post-compile, took 1.7 seconds to fit. A scatter plot comparing staging of the ten-feature PPSI model versus the SuStaIn model can be found in Extended Data 5, as can a cross-tabulation of their subtype assignments.

We then tested the association between each model’s subtyping outputs to the categorical receptor values. The dataset was reduced to records with Positive or Negative receptor values for this specific analysis. Chi-squared tests were used to analyze a contingency table of each model’s subtyping output and the 8 present combinations of receptor values found in the dataset. The 10 feature SuStaIn model’s subtyping showed a strong effect, with χ2 (14, N = 456) = 49.2, p=1e-5. The 10 feature PPSI model also showed a stronger effect with χ2 (14, N = 456) = 116.0, p=1e–17. The 37 feature PPSI model further showed a yet strong effect with χ2 (14, N = 456) = 150.3, p=1e-24.

##### Data Generator Simulation Study

We shall now discuss the multiple simulation studies used to quantify PPSI’s performance across a wide variety of hyperparameter settings and ground-truth conditions. The data generator used in this work produces datasets of cross-sectional observations along generated ground truth polynomial progressions. The generator has many parameters which can be adjusted to produce progressions with varying levels of variable diversity, coefficient magnitude, noise level, sampling bias across stages, class imbalance across subtypes, and more. PPSI models were then fitted to the generated dataset in an attempt to recapture the underlying progression. A full description of the data generation process can be found in Supplementary Materials section 7.

##### Simulation Study 1

In the first study, 100 datasets were generated at random and then fit with a grid of 2,187 combinations of model settings, yielding N = 218,700 models for analysis. In this analysis, hyperparameter settings were explored, their contributions to ground truth progression recovery were quantified, and the impacts of incorrectly specifying subtype count and polynomial order were investigated. The data generator arguments and the grid of hyperparameter settings for Simulation Study 1 can be found in Extended Data section 6.

To explore optimal hyperparameter settings, simple linear models were employed to investigate models’ L1 penalty, maximum scheduled learning rate, number of stages, and minibatch size as predictors for subtyping ARI and staging correlation scores. Ordinary Least Squares (OLS) regression models were fitted to independently investigate staging and subtyping successes (dependent variables) using each individual hyperparameter and a constant term (independents). Full results can be found in Supplementary Materials section 8. A handful of mild impacts were statistically discernible, but the most important was that higher scheduled maximum learning rate positively impacted both staging (B=0.1072, p<0.001) and subtyping (B=0.0551, p<0.001). Also, minibatch size negatively affected subtyping (B=−0.0440, p<0.001) but did not affect staging (B<0.001, p=0.969).

To quantify which dataset characteristics and model settings contributed to subtyping and staging performance, a random forest model (Breiman 2001) was used. The random forest was trained (0.75 training records, 0.25 testing) to predict success metrics based on experiment parameters, model settings, and model hyperparameters. The relative feature importances (mean decrease in impurity, MDI) for predicting success were extracted. The random forest was able to predict subtyping ARI well (R2train=0.92, R2test=0.90), utilizing subtype count discrepancy (MDI=0.38) and subtype class imbalance (MDI=0.36) as the best predictors, with others less useful (MDI<0.06). Staging success was similarly modeled and successful (R2train=0.86, R2test=0.83) utilizing subtype count discrepancy (MDI=0.24) followed by feature usefulness measures and dataset noise level (MDI=0.13–0.11), with others further negligible (MDI<0.05). These results are displayed graphically in Extended Data section 7.

The impact of overestimating and underestimating subtype count and polynomial order were further investigated. Because the results were not well represented using linear regression, a Tukey HSD was used to investigate the impact of subtype count discrepancy, defined as ground-truth number of subtypes (1 to 3) minus modeled number of subtypes (1 to 3), on staging correlation and subtyping ARI. Another Tukey HSD was used to investigate the impact of polynomial order discrepancy, defined as ground-truth order (2 to 4) minus modeled order (2 to 4), on staging correlation and subtype ARI. The outputs of those tests are tabulated in Extended Data section 8. The results show that properly estimating subtype count is more important than properly estimating polynomial order. Further, underestimating subtype count negatively impacts staging and subtyping more than overestimating.

##### Simulation Study 2

All model parameters were held constant while each individual dataset characteristic was tested across a range of values, and the success metrics were then targeted with linear models using the changing characteristic as the regressor. Importantly, these tests were performed using models with the correct subtype count and polynomial order for each dataset. Between 100–300 tests were performed for each target parameter depending on its nature, with revolving subtype counts from 1 to 3 as the parameter climbed or fell, meaning there were between 33 and 100 tests per subtype count, uniformly distributed across the changing target parameter value. The tests for each subtype count were analyzed independently of one another.

The second simulation study quantified staging and subtyping success metrics across a range of adverse conditions, including sample size, sampling bias across stage, class imbalance across subtypes, feature count, feature extremity, feature cluster count, feature cluster heterogeneity, feature cluster size imbalance, and noise level (see Supplementary Materials Section 7 for definitions). Results for simulation study 2 are tabulated in Supplementary Materials section 9. Generally, we can report that staging is more resilient to adverse conditions than subtyping. Dataset sample size did not discernibly impact subtyping or staging performance. Sampling bias across stages and subtype class imbalance were both correlated with lower subtyping performance, but not necessarily with lower staging performance. Feature count, feature extremity (polynomial “curviness”), and feature diversity all impacted both subtyping and staging performance positively. Dataset noise level negatively impacted staging and subtyping performance.

##### Comparison to SuStaIn

As a final measure, PPSI was compared to its predecessor, SuStaIn, in their abilities to recover stages and subtypes from the simulator. To test this, a six-feature, two subtype, second order progression was generated. There was no meaningful difference in staging distribution between subtypes, identical subtype class imbalance, and a moderate-high noise level. 500 records were sampled. PPSI and SuStaIn were fitted to this dataset 20 times each, and their subtyping ARI, staging correlation, and fitting runtime were recorded. SuStaIn averaged a subtyping ARI of 0.773, staging correlation of 0.569, and a runtime of 10 minutes and 35 seconds. PPSI averaged an ARI of 0.768, a correlation of 0.712, and a runtime of 0.62 seconds. Extended Data section 9 contains visualizations for this test.

## Methods

### Algorithm Mathematics

#### Pseudotime - Polynomial Basis Function Expansion

At most simple, pseudotime (t) in a PPSI model is represented as vector of values, ranging from 0 (beginning of a progression) to 1 (end of a progression), and discretized into an equidistant set of “stages”. Number of progression stages hyperparameter (K) defines the number of non-zero stages in a progression. Stage 0 always corresponds to t=0, whereas Stage K always corresponds to t=1. The number of total stages is thus always K+1 due to the additional Stage 0 always included primarily to model the control stage in addition to the K requested progression stages. Below is a general formula to obtain t corresponding to a given stage (k) assuming that K is known:

$$t_{k}=\frac{k}{K}$$

A single linear t column vector represents the simplest possible case of the pseudotime matrix (T) in PPSI, which in a general is $T\in R^{(K+1)\times D}$ (K+1 rows and D columns in size and composed of real numbers). It is obtained by iteratively applying equation 1 to a range of integers from 0 to K:

$$\left[\begin{array}{c} 0\\ t_{1}\\ \vdots \\ t_{\left(k-1\right)}\\ t_{k}\\ t_{\left(k+1\right)}\\ \vdots \\ t_{\left(K-1\right)}\\ 1 \end{array}\right]$$

The significance of obtaining t values corresponding to different stages is because those define the discrete points in the domain of the polynomial (0, 1) where it is evaluated. The T matrix presented above can accommodate only linear monotonic progression (only linearly increasing or linearly decreasing biomarker value with progression). To capture non-linear trends in biomarker progression, we have the option of increasing the maximal polynomial order (D). Thus, for a model with D=2, the T matrix would look like this:

$$\left[\begin{array}{cc} 0 & 0\\ t_{1} & t_{1}^{2}\\ \vdots & \vdots \\ t_{k-1} & t_{k-1}^{2}\\ t_{k} & t_{k}^{2}\\ t_{k+1} & t_{k+1}^{2}\\ \vdots & \vdots \\ t_{K-1} & t_{K-1}^{2}\\ 1 & 1 \end{array}\right]$$

A more general example looks like this (d specifies some specific polynomial degree less than D):

$$\left[\begin{array}{ccccc} 0 & \cdots & 0 & \cdots & 0\\ t_{1} & \cdots & t_{1}^{d} & \cdots & t_{1}^{D}\\ \vdots & \; & \vdots & \; & \vdots \\ t_{k-1} & \cdots & t_{k-1}^{d} & \cdots & t_{k-1}^{D}\\ t_{k} & \cdots & t_{k}^{d} & \cdots & t_{k}^{D}\\ t_{k+1} & \cdots & t_{k+1}^{d} & \cdots & t_{k+1}^{D}\\ \vdots & \; & \vdots & \; & \vdots \\ t_{K-1} & \cdots & t_{K-1}^{d} & \cdots & t_{K-1}^{D}\\ 1 & \cdots & 1 & \cdots & 1 \end{array}\right]$$

Beyond third order polynomial it seems there is little reason to add additional degree. At that point, the basis-expansion matrix can be used to implement a cubic polynomial spline, which fits a piecewise cubic polynomial function with hinges, closer in nature to the original SuStaIn approach that we built on, but with added flexibility of being able to model arbitrary progression shapes. This advanced future expansion of our novel method, however, is beyond the scope of present discussion.

#### Polynomial Progression Modelling - Polynomial Coefficient Parameters

The aspect of the model trained by gradient descent are the polynomial coefficients ($\theta_{d,p}$), a single subtype matrix of which $\Theta_{s}\in R^{D\times P}$ (is of size D rows by P columns and composed of real numbers).


$$\left[\begin{array}{ccccc} \theta_{1,1} & \cdots & \theta_{1,p} & \cdots & \theta_{1,P}\\ \vdots & \; & \vdots & \; & \vdots \\ \theta_{d,1} & \cdots & \theta_{d,p} & \cdots & \theta_{d,P}\\ \vdots & \; & \vdots & \; & \vdots \\ \theta_{D,1} & \cdots & \theta_{D,p} & \cdots & \theta_{D,P} \end{array}\right]$$

For multiple subtypes, we extend the parameters matrix into the 3rd dimension: $\Theta \in R^{D\times P\times S}$ (is of size D rows by P columns by S subtype 3D layers and composed of real numbers). S represents the maximal allowed subtype. The cardinality of (number of real numbers in) Θ represents the number of trainable model parameters, and is thus multiplicatively proportional to the maximal number of polynomial degree allowed, number of features included in the model, and the highest number of subtypes allowed.

#### Progression Feature Polynomial Evaluation – Matrix Multiplication

The first and central computation in PPSI is the matrix multiplication:

$$M_{s}=T\Theta_{s}$$

It essentially evaluates each of the feature polynomials at various stage t-pseudotime values. Thus $M_{s}\in R^{(K+1)\times P}$ (K+1 rows and P columns in size and composed of real numbers). The representation below for a 3rd degree polynomial example might give better intuition as to what the matrix multiplication is doing:

$$\left[\begin{array}{ccccc} 0 & \cdots & 0 & \cdots & 0\\ \left(\theta_{1,1}t_{1}+\theta_{2,1}t_{1}^{2}+\theta_{3,1}t_{1}^{3}\right) & \cdots & \left(\theta_{1,p}t_{1}+\theta_{2,p}t_{1}^{2}+\theta_{3,p}t_{1}^{3}\right) & \cdots & \left(\theta_{1,P}t_{1}+\theta_{2,P}t_{1}^{2}+\theta_{3,P}t_{1}^{3}\right)\\ \vdots & \vdots & \; & \; & \vdots \\ \left(\theta_{1,1}t_{K}+\theta_{2,3}t_{K}^{2}+\theta_{3,1}t_{K}^{3}\right) & \cdots & \left(\theta_{1,p}t_{K}+\theta_{2,p}t_{K}^{2}+\theta_{3,p}t_{K}^{3}\right) & \cdots & \left(\theta_{1,P}t_{K}+\theta_{2,P}t_{K}^{2}+\theta_{3,P}t_{K}^{3}\right) \end{array}\right]$$

Plotting each column vector in the $M_{s}$ matrix with respect to stage reveals the point estimate of expected biomarker value at each of the stages for a given subtype. In a simplified notation, the entries of $M_{s}$ can be presented such:

$$\left[\begin{array}{ccccc} 0 & \cdots & 0 & \cdots & 0\\ \mu_{1,1} & \cdots & \mu_{1,p} & \cdots & \mu_{1,P}\\ \vdots & \; & \vdots & \; & \vdots \\ \mu_{K,1} & \cdots & \mu_{K,p} & \cdots & \mu_{K,P} \end{array}\right]$$

In a similar manner to Θ,M is simply a higher dimensional representation of $M_{s}$ where the additional dimension allows each subtype to have its own set of multivariate biomarker value predictions by stage. Thus: $M\in R^{(K+1)\times P\times S}$

### Data Matrix

The input to our algorithm (X):

$$\left[\begin{array}{ccccc} X_{1,1} & \cdots & X_{1,p} & \cdots & X_{1,P}\\ \vdots & \; & \vdots & \; & \vdots \\ X_{n,1} & \cdots & X_{n,p} & \cdots & X_{n,P}\\ \vdots & \; & \vdots & \; & \vdots \\ X_{N,1} & \cdots & X_{N,p} & \cdots & X_{N,P} \end{array}\right]$$

N is number of total data points, n is specific data point index, P is maximal feature number, p is some specific feature index.

#### Residualization-based Progression Feature Normative Z-scoring

Though our algorithm has the potential of being applicable in many different domains, the principal application of interest to us is for data-driven modelling of disease progression. Thus, pre-processing of progression values in our case relies on the assumption that there is some control “normal” value for each of the biomarkers that we would expect to see for observations that are not yet undergoing the progression under investigation (i.e. control patients), but that might vary as a function of multiple potentially confounding variables (i.e. normal changes related to aging and sex). We utilize a strategy previously described by a Python implementation of the original SuStaIn algorithm (Aksman et al. 2021), and subsequently applied in our prior work on cholinergic system progression in Parkinson’s disease (Bohnen et al. 2025).

First, a confounder model is fitted for a progression feature (Y), predicting it from a set of “confounder” regressors of interest (β) within a sample of “controls” (observations which we know not to be a part of the progression being studies). For the example below, we will stick to a scenario widely applicable in biomedical sciences, of covarying out the effect of normal aging and sex-specific differences:

$$Y∼\beta_{\mathrm{Age}\;}X_{\mathrm{Age}\;}+\beta_{\mathrm{Sex}\;}X_{\mathrm{Sex}\;}+\varepsilon$$

Within this formula, ε represents the model error or residual. To obtain Z-scores relative to the control population after controlling for confounder variables of interest (Z; adjusted for effect of age & sex among controls in this example), the feature value for observations that we know to be undergoing the progression of interest is predicted by the “control” model ($\hat{Y}$), the prediction is subtracted from the actual observed feature value (Y), and then divided by the standard deviation of the model residuals $\left(s_{\varepsilon }\right)$:

$$Z=\frac{Y\;-\;\hat{Y}}{s_{\varepsilon }}$$

Thus, even though the unadjusted progression feature value (Y) might naturally vary as a function of age and sex among controls, Z-scored and adjusted progression feature value (Z) will always represent the value which we would expect from “control” observations for a given age and sex. Same logic is easily extended to other potential confounder variables of interest. For example, if lifestyle factors like smoking and drug consumption are known to systematically modify the progression feature value in a manner independent of a progression being studied, there might be reason to include them in the Z-scoring model, to reduce the noise variance in the features from which we are attempting to learn a progression. In all subsequent discussion of the data matrix (X), it is assumed that the values of the matrix are derived from a Z-scoring procedure as described above.

#### Single Observation Likelihood Calculation at a given Stage & Subtype

First, let’s consider a single observation row vector ($X_{n}$)

$$\left[\begin{array}{lllll} X_{1} & \cdots & X_{p} & \cdots & X_{P} \end{array}\right]$$

To calculate the likelihood of a single observation at a given subtype (s) and stage (k) is a cumulative product over observed feature value likelihoods given the value expected of them based on the evaluated polynomial matrix (M).


$$\phi_{s,k}\left(X_{n}\right)=\prod_{p=1}^{P}PDF\left(\mu_{k,p,s},\sigma ,X_{n,p}\right)$$

Where σ∈|R| (is a scalar, real, positive value) and is a fixed hyperparameter setting the standard deviation size on each normal distribution of which we evaluate the probability density function (PDF) based on the mean obtained from the M entry at a given stage, feature, and subtype. Keeping σ fixed allows us to implement an optimization on log-likelihood calculation which results in ~13 times speed-up in model training, discussed in a later section.

#### Optimal Subtype and Stage Assignment for a given Observation

$\Phi_{n}\in R^{S\times (K+1)}$ is a matrix of $\phi_{s,k}$ values, representing the likelihood of a given observation (n) over all the stages and subtypes. To obtain a given individual’s (n) maximal likelihood subtype $\left(s_{ML}\right)$ and stage $\left(k_{ML}\right)$:

$$s_{ML},k_{ML}=\underset{\forall s\forall k}{argmax}\;\Phi_{n}$$

∀s signifies “all subtypes”, and ∀k signifies “all stages”. We are finding the indices corresponding to highest likelihood across all subtypes and all stages.

#### Model Loss Function

To calculate the likelihood of the whole dataset given our current model parameters, we take the cumulative product of these maximal likelihoods obtained across all our observations:

$$ℒ(X|\Theta )=\prod_{n=1}^{N}\underset{\forall s\forall k}{max}\;\Phi_{n}$$

We implement $L_{1}$ loss to regularize our model trainable parameters by taking the sum of absolute valued parameter values:

$$L_{1}=\sum_{S=1}^{S}\sum_{p=1}^{P}\sum_{d=1}^{D}|\theta |$$

The final loss function is a linear combination of negative model likelihood and $L_{1}$ loss scaled by $L_{1}$ penalty hyperparameter (λ):

$$L(\Theta )=-ℒ(X|\Theta )+\lambda L_{1}$$

#### Model Training via Gradient Descent

The model parameters (Θ), aka polynomial coefficients, are trained to maximize the likelihood of the data given the model (by minimizing negative likelihood) while keeping model complexity minimal (by minimizing the scaled $L_{1}$ loss). Parameters are updated by taking the derivative of the loss function with respect to current parameters:

$$\dot{\Theta }=\frac{d}{d\Theta }L(\Theta )$$

Once the derivative is obtained, it is multiplied by the learning rate (η) and subtracted from current parameter values (Θ) to yield the updated parameter values $\left(\hat{\Theta }\right)$:

$$\hat{\Theta }=\Theta -\eta \dot{\Theta }$$

This process is repeated iteratively until user-defined maximal number of iterations is reached. The learning rate is dynamically modulated based on iteration number with an annealing-based learning rate scheduler, to accelerate convergence and yield more stable model estimates (Nakamura et al. 2021).

#### Theoretical Time Complexity

The time complexity to run a single iteration of the PPSI loss function is proportional to the maximal number of subtypes (S), total number of stages (K), number of progression features (P), and number of observations within a dataset (N). Thus, the theoretical time complexity of the algorithm with respect to the hyperparameters and number of observations is O(SKPN), meaning the runtime of our algorithm will increase linearly if either S,K,P, or N is increased while the others are held constant.

#### Implementation & Optimization

Evaluating the probability density function (PDF) usually returns a relatively small floating-point number. Cumulative multiplication of many small floating-point numbers yields a very small number, with the number of decimal point needed to represent it above what is allowed by single precision floating point data type implemented in most programming languages, which leads to “arithmetic underflow” errors. The solution to this problem lies in the application of logarithms, and their properties with respect to representing very small numbers and transforming multiplication into addition. Furthermore, the assumption that we make about σ being fixed across subtype-stage specific normal distributions allows us to implement a memoization-based optimization for log-PDF computation. We derive the optimized log-PDF implementation for fixed σ applications from the original PDF of a normal distribution formula below:

$$PDF(\mu ,\sigma ,x)=\frac{1}{\sigma \sqrt{2\pi }}e^{-\frac{{\left(x\;-\;\mu \right)}^{2}}{2\sigma^{2}}}$$

The simplification is performed by considering the parts of the equation that can be evaluated once, in advance only with knowledge of σ alone. This yields two pre-computable parameters, the coefficient (α) and the exponent denominator (β):

$$\alpha =\frac{1}{\sigma \sqrt{2\pi }}\;\beta =2\sigma^{2}$$

These values are pre-computed once and stored in stack memory. The following simplified formula can then be used to compute the PDF:

$$PDF(\mu ,x)=\alpha e^{-\frac{(x\;-\;\mu {)}^{2}}{\beta }}$$

If log PDF is what we’re interested in, we take the natural log of both sides, and are left with an even simpler formula:

$$log\;PDF(\mu ,x)=\alpha -\frac{(x\;-\;\mu {)}^{2}}{\beta }$$

Our preliminary findings show that this fixed σ log PDF approach leads to a 13 times speed-up to a core computation which is performed S×K×P×N times per single algorithm iteration, thus making its application more scalable to larger datasets, problems that involve many progression features, or that require a large number of stages or subtypes to be modelled.

The only change to the underlying algorithm required to accommodate the use of log PDF instead of the PDF is the substitution of cumulative addition in place of cumulative multiplication for stage and subtype likelihood value:

$$\phi_{s,k}\left(X_{n}\right)=\sum_{p=1}^{P}log\;PDF\left(\mu_{k,p,s},\sigma ,X_{n,p}\right)$$

And the same for the overall model likelihood:

$$ℒ(X|\Theta )=\sum_{n=1}^{N}\underset{\forall s\forall k}{max}\;\Phi_{n}$$

### Recommended Workflow

The authors have developed a workflow for practitioners applying PPSI to a preprocessed dataset. It is an iterative process wherein users fit a range of PPSI models, examine the results mechanically, decide which combination of subtype count and polynomial order to use, potentially reduce the number of features to those most informative, and then leverage included plotting functions for subject matter experts to evaluate the fitted model’s explanations of their data. This process is then repeated until the obtained model is sound and useful.

#### Cross-validated Subtype Sweep

As PPSI is an unsupervised algorithm, selecting the correct number of subtypes is an important challenge during the modeling process. Again, the first step is to fit a range of models and assess them mechanically. We do this by performing a “sweep” across subtype counts and polynomial orders. Much like a clustering algorithm might be iteratively fitted with an increasing number of clusters in an attempt to “find the elbow” in a success metric (Robert Tibshirani, Walther, and Hastie 2001), PPSI is fitted on many model settings and then six different metrics are produced to shed light on the results. By default, first, second, and third order polynomials are evaluated across one, two, three, and four subtype models, with ten PPSI models generated per hyperparameter combination and trained on different cross-validation folds (120 models total). The results of the sweep are then evaluated using six metrics the authors have developed, each providing insight into the behavior of the model and the nature of the dataset (Supplementary Material 5). After evaluating these metrics, an estimation of justified polynomial order and subtype count are selected

#### Multiple Evaluation Metrics

Prior methods (SuStaIn), rely on the distribution of single evaluation metrics (model log likelihood) for choosing the appropriate number of subtypes (Young et al. 2018). We believe that inferring longitudinal progressions from cross-sectional data is inherently an ill-posed problem, in that multiple solutions may provide equivalent fit, meaning that a more holistic approach is required. PPSI subtype sweep includes six metrics by default, which we found to be predictive of the correct number of subtypes in simulation studies, or otherwise informative about the model. These metrics are described in greater detail in Supplementary Materials section 5, but briefly presented here.

Stage distribution reflects the standard deviation of the number of observations assigned to each stage and quantifies how evenly the data is distributed across the inferred progression stages (lower is better). Evaluation distance captures how differentiated evaluated polynomial curves are from one another across subtypes, higher values mean more distinct subtypes, lower values mean more similar subtypes. Silhouette score captures how well observations are classified on their subtype and stage relative to their next best subtype and stage (lower indicates a smoother or homogenously subtyped model). Correlation comparison captures the correlation between subtype-specific feature covariances estimated from data vs. covariances estimated from evaluations of subtype-specific polynomial equations (higher is better). AIC reflects model quality scaled by model complexity (standard definition). Euclidean R-squared represents the distance between the multidimensional data points and the subtype polynomial line evaluated at that subtype relative to distance between the means of each feature and the datapoint (very close to standard R-squared definition). The distribution of these model evaluation metrics obtained from cross-validated subtype sweep is generally visualized as a boxplot, showcased in Extended Data sections 1 and 3.

#### Feature Reduction

Feature reduction may now be performed if desired, and a methodology similar to Recursive Variable Elimination is proposed (Guyon et al. 2002). Described further in Supplementary Material section 10, this method repeatedly performs inference using a fitted model while recursively withholding one feature at a time, excluding that feature from use in inference. The proportion of records which change subtype or stage from their original full-featured assignment indicate the importance of the withheld feature. A cutoff value may be set for the percentage of changed assignments for each feature, and further modeling may be performed using only those features above the specified importance cutoff. We demonstrate this process in our breast cancer study.

#### Qualitative Model Evaluation

From the pool of models fitted with the chosen subtype count and polynomial order, a single representative model may be selected for further modeling work. Subject matter experts may now evaluate the model’s explanation of their data using purpose-built plotting functions included in the PPSI code package. With these plots, subject matter experts may display progression polynomials, isolate features and view the distribution of data around them, plot continuous and categorical variables which were not included in training, assess individual records’ staging and subtyping likelihoods across stages and subtypes, expose how records change in stage and subtype as additional subtypes are added, and more. We showcase these plots in Extended Data section 10.

#### External Validation

Finally, the model may be externally validated using unseen data. If longitudinal data is available, the model can be tested to ensure that, when trained only on the chronologically first observation set, the proceeding observations progress forward in stage and remain assigned to the same subtype. Secondly, if other pertinent information is withheld from the modeling process, especially measures which are already explored in previous subtyping efforts, it can be used to examine whether the model is recapitulating understood subtyping patterns.

## Extended Data

**Extended Data 1:**
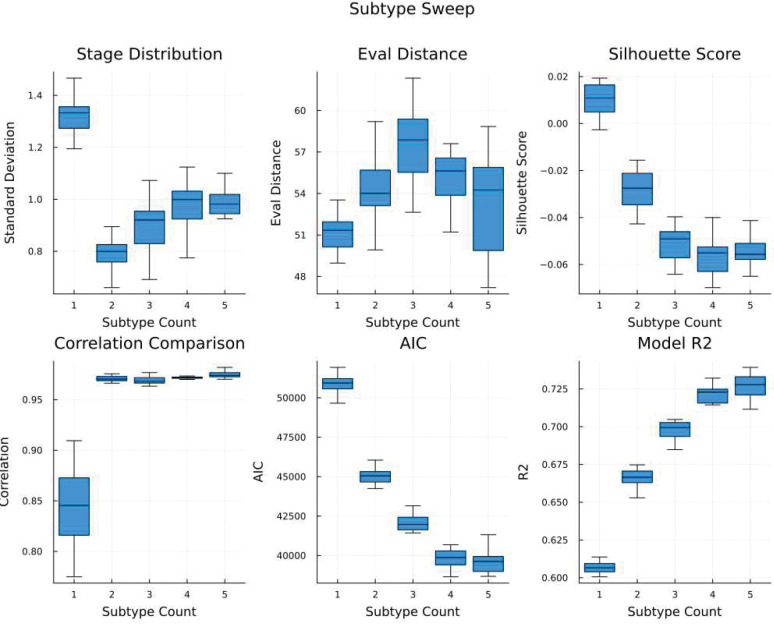
ADNI Subtype Sweep

**Extended Data 2:**
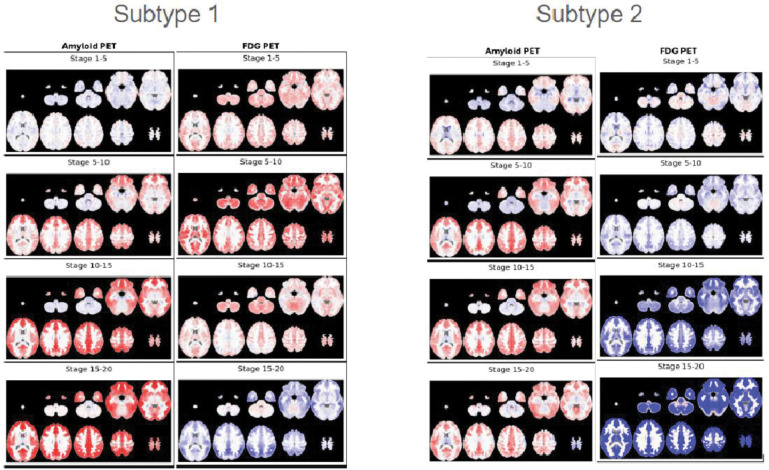
ADNI Subtypes

**Extended Data 3:**
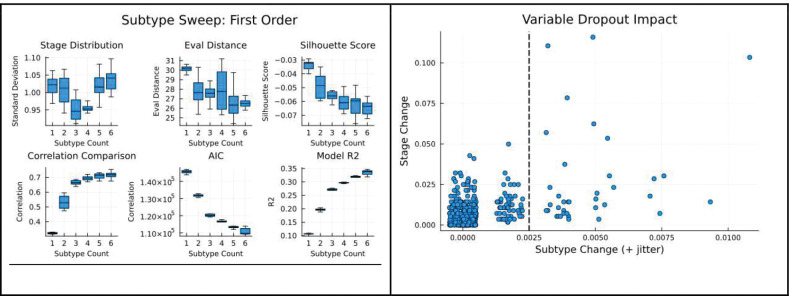

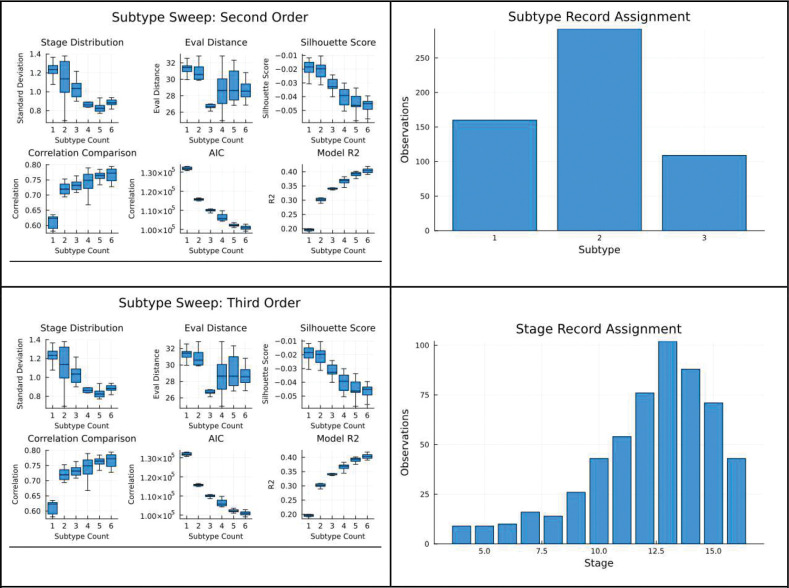
Extended Cancer Modeling Plots Visualizations of 32 variable subtype sweeps and the selected model for the breast cancer result. Top left: A sweep of first order models, 1–6 subtypes. Center left: A sweep of second order models, 1–6 subtypes. Bottom left: A sweep of third order models, 1–6 subtypes. Top right: Variable importances resulting from the Dropout Report, with the cutoff visualized as a black dotted line. Center right: The number of records assigned to teach subtype in the final model. Bottom right: The number of records assigned to each stage in the final model.

**Extended Data 4:**
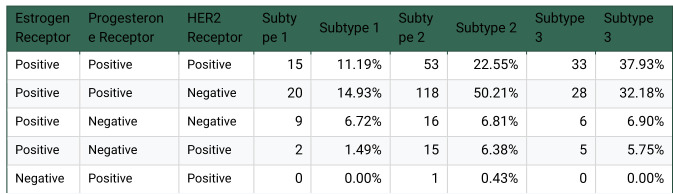

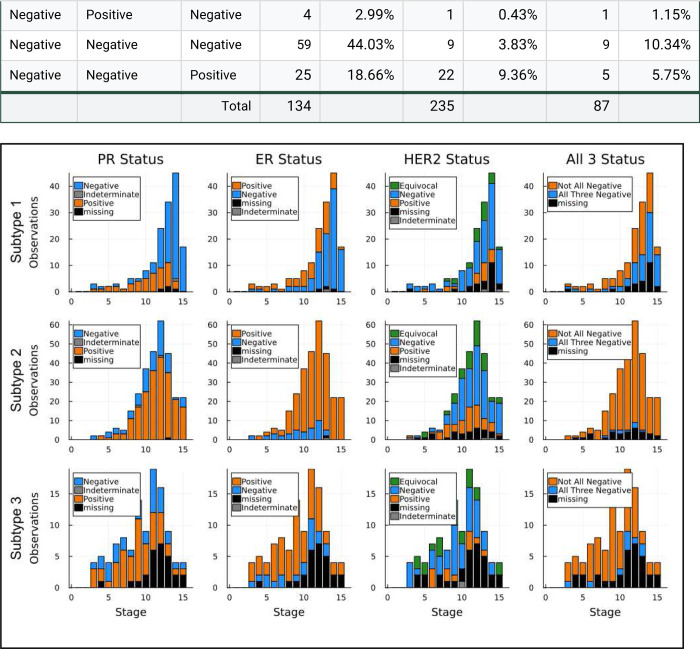
Cancer Outcomes

**Extended Data 5:**
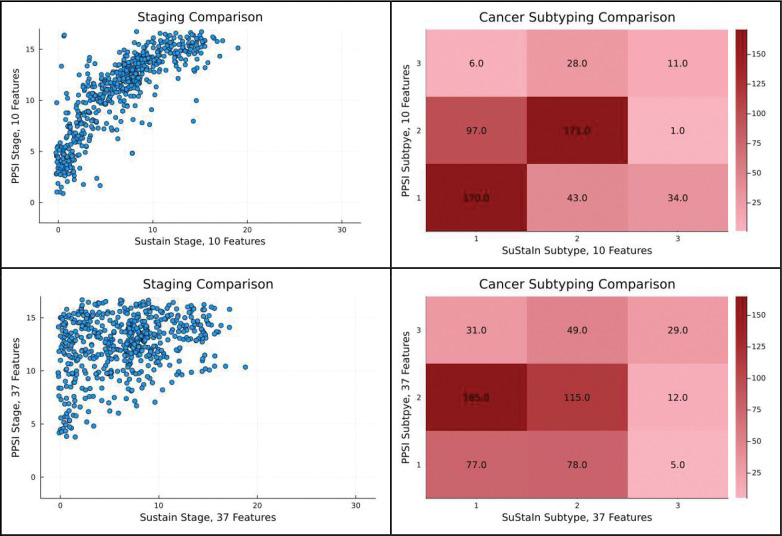
PPSI vs SuStaIn - Cancer Staging and Subtyping Top left: A scatter plot comparing SuStaIn’s staging assignments versus PPSI’s staging assignments for the 10 variable cancer proteins dataset, jitter added. Top Right: A heatmap crosstabulation comparing SuStaIn’s subtype assignments versus PPSI’s subtype assignments for the 10 variable cancer proteins dataset. Bottom left: A scatter plot comparing 37-feature SuStaIn’s staging assignments versus 37-feature PPSI’s staging assignments for cancer proteins dataset, jitter added. Bottom Right: A heatmap crosstabulation comparing 37-feature SuStaIn’s subtype assignments versus 37-feature PPSI’s subtype assignments for the cancer proteins dataset.

**Extended Data 7:**
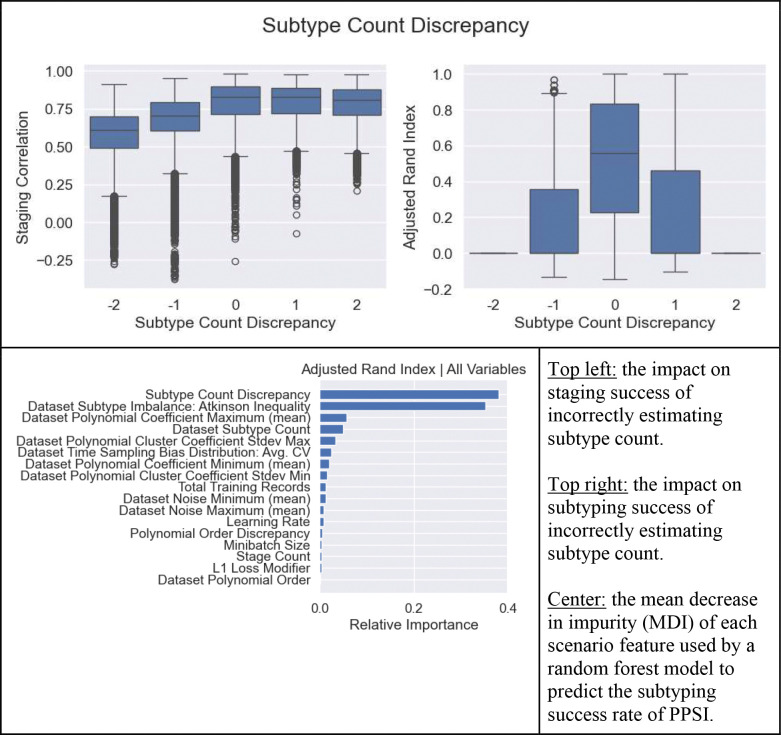

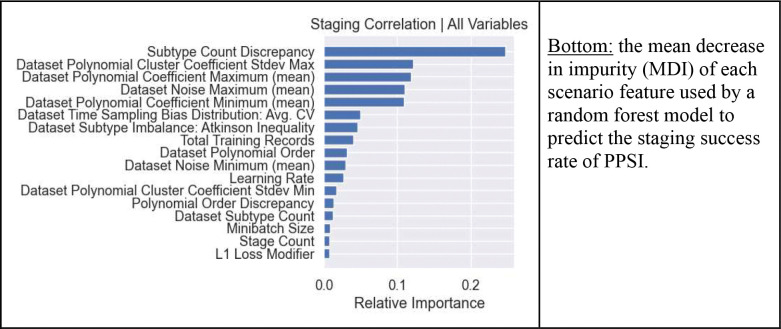
Simulation Study 1 Plots

**Extended Data 9:**
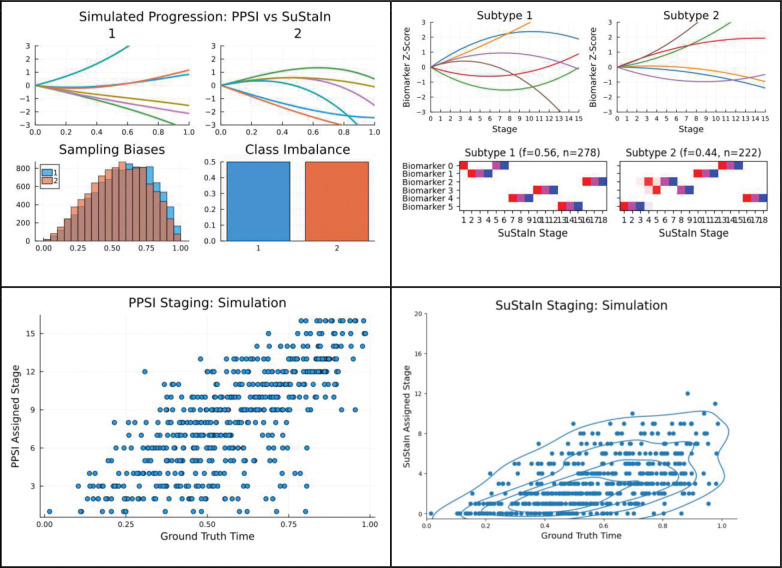
PPSI vs SuStaIn Comparisons - Simulation Datasets Top left: A simulated progression dataset used to test PPSI and SuStaIn side-by-side. The simulation is of a two subtype, 2nd order polynomial dataset. Top right: PPSI’s captured progression (top) and SuStaIn’s captured progression (bottom) of the simulation. Bottom left: PPSI’s captured staging compared to simulated ground-truth. Bottom right: SuStaIn’s captured staging compared to simulated ground-truth.

**Extended Data 10:**
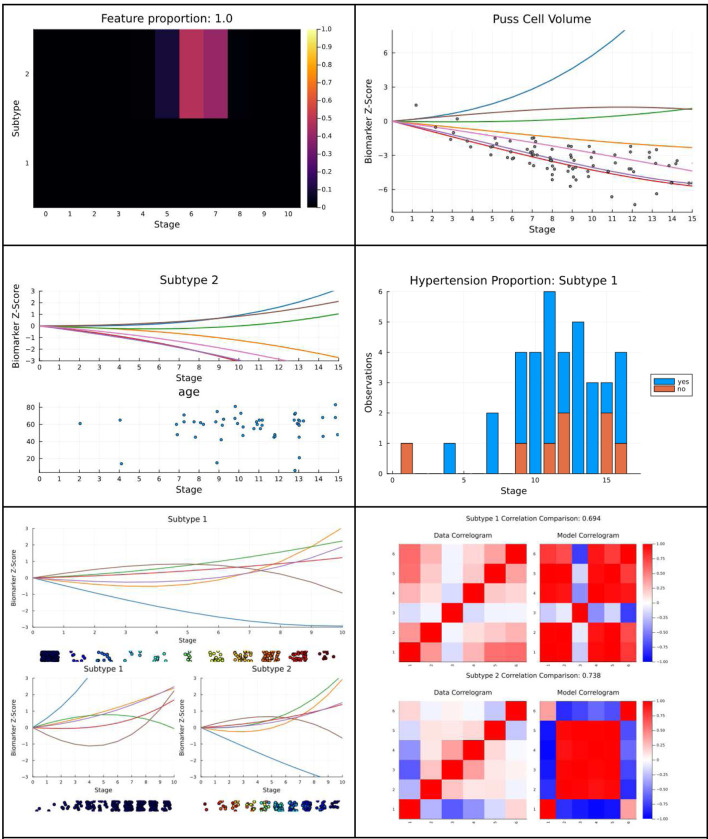
Subject Matter Expert Plots A sample of plots from the PPSI subject matter expert plotting suite. Solutions displayed originate from either PPSI data simulations (sim) or the famous public Chronic Kidney Disease (ckd) dataset (Eswaran 2015). Top left: likelihood distribution of a single record placement across subtype and stage (sim). Top right: an overlaid scatter progression plot (ckd). Center left: a plot of an external (non-modeled) continuous variable (ckd). Center right: a plot of an external categorical variable (ckd). Bottom left: A subtype transition plot from 1–2 subtype solutions (sim). Bottom right: a model vs data correlation comparison (sim).

**Extended Data 6:** Simulation Study 1 Experiment Parameters

Dataset Generator Parameters		
Parameter	Values (or range)	Description
means	1 to 4	Range of means for polynomial clusters. Higher values create curvier polynomial lines.
stdev_min	0.2 to 0.5	Minimum and maximum standard deviation of polynomial coefficient clusters. Higher means more diverse clusters. Wide range means varied clusters.
stdev_max	stdev_min to 1.5	
noise_min	0.2 to 1.5	Minimum and maximum noise coefficient for polynomial
noise_max	noise_min to 1.5	
n_subtypes	[1, 2, 3]	Ground truth number of subtypes
dimensions	[1, 2, 3]	Ground truth polynomial order (also number of dimensions in a coefficient cluster)
n_polynomials	5 to 50	Number of polynomials / columns (features)
n_clusters	4 to n_polynomials	Ground truth number of polynomial clusters
sampling_bias_minmax	(1.0, 4.0)	Range of possible alpha and beta values for Beta distributions to describe a subtype’s sampling bias
polydist_imbalance_index	(0.1)	Target Atkinson inequality for how evenly polynomials are assigned to coefficient clusters
subtype_class_imbalance _index	(0.1)	Target Atkinson inequality for subtype membership (class imbalance)
record_count	60 to 1000	Number of records drawn from the generated progression
Model Hyperparameters		
Hyperparameter	Values (or range)	Description
Learning Rate	[0.1, 0.01, 0.001]	Coefficient change per iteration. This value was the maximum used in the learning rate scheduler.
L1 Penalty	[0.05, 0.1, 0.15]	Penalizes model for sum of coefficients. Higher L1 causes straighter lines. (regularization)
Number of Stages	[10, 15, 20]	Number of possible stages to which participants can be assigned from 0 to 1
Minibatch Size	[0.1, 0.2, 0.3]	Proportion of records to train with per iteration. (regularization)
Polynomial Order	[2, 3, 4]	Ground truth polynomial order (also number of dimensions in a coefficient cluster)
Number of Subtypes	[1, 2, 3]	Number of ground truth subtypes
Sigma	[0.5, 1.0, 1.5]	Standard deviation of the normal distribution placed around the polynomials at each stage, used in calculating PDF

**Extended Data 8:** Simulation Study 1 Statistical Results

Model of Staging Correlation Scores ~ Subtype Discrepancy		
Subtype Discrepancy	Mean Staging Correlation	St. Dev Staging Correlation
−2	0.58	0.168
−1	0.68	0.161
0	0.79	0.139
1	0.79	0.126
2	0.78	0.125

**Table:** Tukey HSD: Subtyping ARI ~ Subtype Discrepancy

Group 1	Group 2	Mean Diff	P-Adj	Reject
−1	0	0.1153	0	TRUE
0	1	−0.1216	0	TRUE

*-2 and 2 discrepancy omitted because single-subtype vs multiple subtype ARI is undefined

**Table:** Tukey HSD: Subtyping ARI ~ Polynomial Order Discrepancy

Group 1	Group 2	Mean Diff	P-Adj	Reject
−2	−1	0.0628	0	TRUE
−1	0	−0.0044	0.3951	FALSE
0	1	0.0289	0	TRUE
1	2	−0.0432	0	TRUE

**Table:** Tukey HSD: Staging Correlation ~ Subtype Discrepancy

Group 1	Group 2	Mean Diff	P-Adj	Reject
−2	−1	0.0947	0	TRUE
−1	0	0.1133	0	TRUE
0	1	0.0009	0.8228	FALSE
1	2	−0.0101	0	TRUE

**Table:** Tukey HSD: Staging Correlation ~ Polynomial Order Discrepancy

Group 1	Group 2	Mean Diff	P-Adj	Reject
−2	−1	−0.0026	0.196	FALSE
−1	0	−0.0038	0.0004	TRUE
0	1	−0.0046	0	TRUE
1	2	−0.0097	0	TRUE

## Supplementary Material

Supplement 1

Additional Information

Supplementary Information is available for this paper.

## Figures and Tables

**Figure 1.**
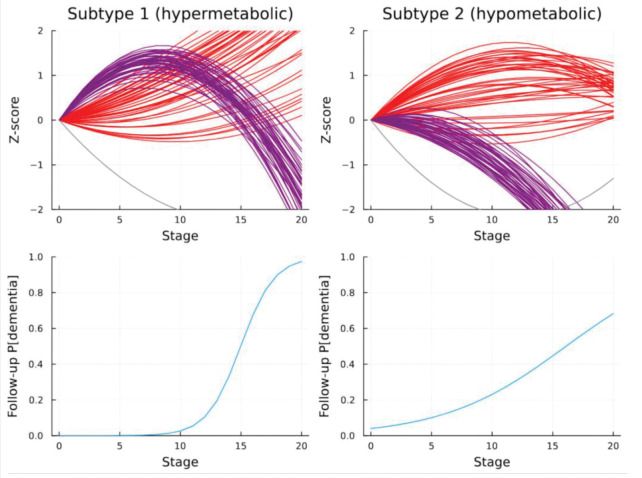
Top row: polynomial curve plots corresponding to cerebral FDG PET uptake (glucose metabolism) biomarkers in purple, cerebral AV45 PET uptake (amyloid deposition) in red, and CSF ABETA42 in gray. Bottom row: logistic regression predicted probability of follow-up (~2 year) dementia status given baseline stage by subtype for patients without dementia at baseline (only mild cognitive impairment). The hypermetabolic subtype appears more resilient to future development of dementia at earlier stages when the hypermetabolic process is actively expressed, however the probability of future emergence of dementia rapidly increases as the hypermetabolic process peaks, immediately preceding a sharp decline in cerebral metabolism and an increase in brain amyloid deposition. The hypometabolic subtype appears more susceptible to have dementia at 2-year follow-up, even at earlier stages, but the probability of observing dementia at 2-year follow-up rises more gradually with increased baseline stage. For visualizations of these changes in the brain, please see Extended Data section 2.

**Figure 2.**
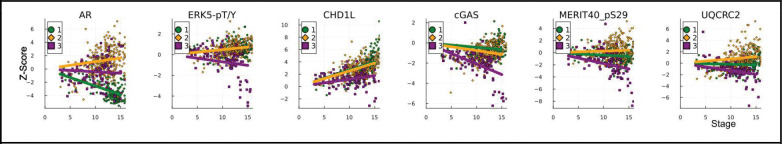
Each subtype’s cancer protein interaction with stage is assessed via linear model. Modeled with intercept at 0 and contrasting against subtype 2 (most numerous.) Linear model results are tabularized in Table 2.

**Table 1.** A summarization of performed comparisons between SuStaIn and PPSI.

Studies / Algorithms	SuStaIn	PPSI
Alzheimer’s: Longitudinal Validations	82% subtype consistency 80% staging monotony 59% staging progression *Unidirectional disease progression*	80% subtype consistency 84% staging monotony 60% staging progression *Bidirectional disease progression*
Breast Cancer: Subtyping Validations	10 features: *χ*^2^ (14, N = 456) = 52.78, p=1e^−5^ runtime = **4.56 hours** *Only reduced variable set practically usable*	10 features *χ*^2^ (14, N = 456) = 116.0, *p*=1e^−17^ runtime = **1.65 seconds** 32 features: *χ*^2^ (14, N = 456) = **150.3, *p*=1e^−24^** runtime = **4.61 seconds** *482 protein variables usable at once*
Simulations: Performance Comparison	0.773 subtyping ARI 0.569 staging correlation runtime = 10.5 minutes average	0.768 subtyping ARI 0.712 staging correlation runtime = 0.62 seconds average

**Table 2.** Results of the linear models depicted in Figure 2. Effects of subtype without stage interaction not shown.

Protein	Subtype 1 * Stage		Subtype 2 * Stage		Subtype 3 * Stage	
	*p*	B [CI_95_]	*p*	B [CI_95_]	*p*	B [CI_95_]
AR	<1e-18	−0.617 [−0.746, 0.488]	<1e-4	−2.144 [−3.17, −1.12]	0.026	−0.151 [−0.285, −0.018]
BMK1-Erk5_pT218_Y220	0.0021	−0.085 [−0.139, −0.031]	0.0002	−0.807 [−0.124, −0.378]	<1e-31	−0.358 [−0.-413, −0.302]
CHD1L	<1e-7	0.277 [0.181, 0.374]	0.585	−0.214 [−0.982, −0.555]	<1e-07	−0.286 [−0.386, −0.186]
cGAS	0.052	−0.061 [−0.122, 0.001]	<1e-29	−2.989 [−3.475, −2.501]	<1e-28	−0.387 [−0.450, −0.324]
MERIT40-pS29	<1e-4	−0.211 [−0.313, −0.110]	0.003	−1.220 [−2.030, −0.412]	<1e-19	−0.506 [−0.611, −0.401]
UQCRC2	<1e-5	−0.243 [−0.343, −0.144]	<1e-18	−3.760 [−4.551, −2.969]	<1e-34	−0.691 [−0.794, −0.588]

## Data Availability

The data that supports the findings of this study are available from the corresponding author upon request.

## Code Availability

All code which was relevant to the implementation of custom methods utilized in the present analysis is made available as a CodeOcean capsule: link

The PPSI package will be made available to the public at: https://github.com/augustvanhout/PPSI
