# Supplementary material for: The Polynomial Progression Subtype Inference Algorithm
Source: Res Sq. 2025 Oct 7:rs.3.rs-7199106. Preprint. [Version 1] doi: 10.21203/rs.3.rs-7199106/v1 (PMC12632589; doi:10.21203/rs.3.rs-7199106/v1)
Supplement: Supplement 1 [file NIHPPrs7199106v1-supplement-1.pdf]

Supplementary Material

Supplementary Material 1: PPSI Mechanics Explanation

The model represents every feature as its own polynomial line through time. For each feature the model will use in fitting, a polynomial equation of order d is initialized by drawing d coefficients from $N(0, 1)$, each with an intercept term of 0. The intercept term is fixed at 0 to ensure that all feature progressions begin from the mean of normative value distribution (mean of the control observations, or simply 0 in data not suitable for Z-scoring).

The data-driven time axis (progression time, pseudotime) is discretized into evenly spaced stages. At each stage, the evaluation of each feature's polynomial equation yields the first moment (location) of a normal distribution, with a fixed variance (hyperparameter "sigma") set at an arbitrary value, often 0.5 or 1.0. To estimate the likelihood that a single record belongs to a given stage, we take the probability density function (PDF) of each feature's value against their corresponding normal distributions, and take a cumulative product of these feature PDFs to derive the record's likelihood of belonging to that stage.

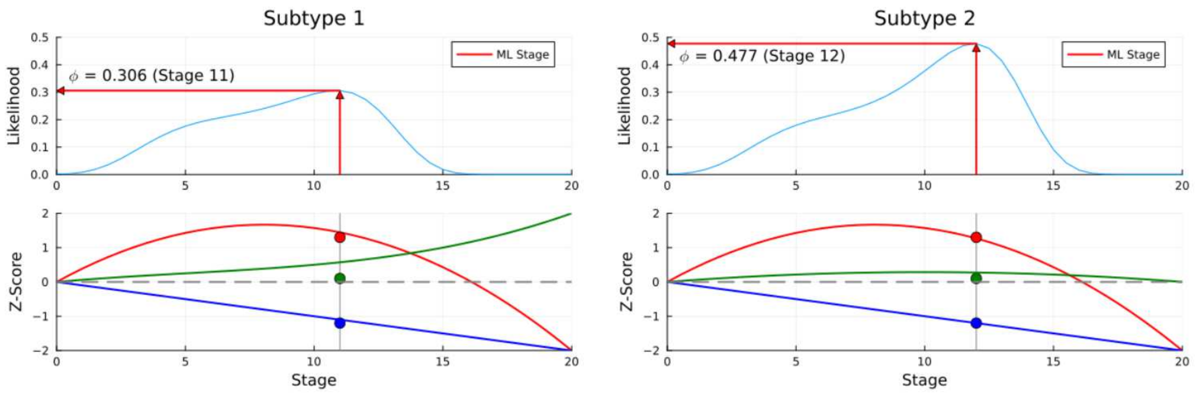

A visualization of stage and likelihood assignment. A single record (red, green, blue dots) is fitted across all stages for all subtypes (bottom plots). The likelihood of these assignments is calculated (top plots) and the maximal likelihood stage and subtype are determined (subtype 2, stage 12).

We perform this evaluation on all stages and select, for that record, the most likely stage. That is to say, we place a record at the stage where their values are most likely to have occurred given normal distributions around the progression's polynomial lines. The record's nominal likelihood is the evaluated likelihood at the maximal likelihood stage. A model's likelihood is the cumulative product of all records' nominal likelihoods, and is used as a loss term to fit the model. Finally, an L1 regularization (R. Tibshirani 1996) is applied, penalizing the model for the absolute value of its coefficients, incentivizing the model to create simple polynomial shapes. PPSI model training entails incremental adjustment of polynomial coefficients to maximize the model likelihood via gradient descent. In this way, the model descends down the error gradient towards a simple set of polynomials which make the data most likely. In practice, this is performed in small batches of randomly selected records, normally 10-30% of the dataset per iteration, called minibatch training (Bottou 2010). This lowers computational intensity and combats model overfitting. PPSI also employs a learning rate scheduler to reduce computation workload and improve model stability (Nakamura et al. 2021).

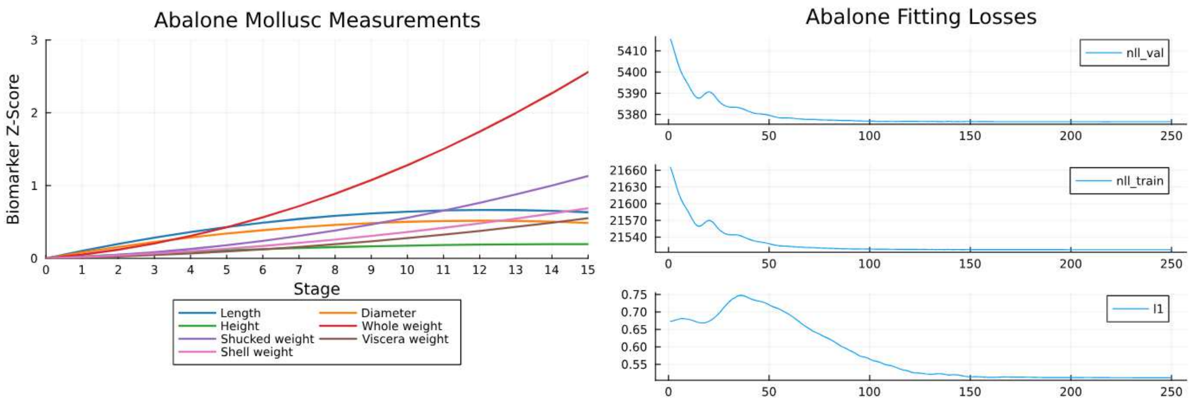

A simple 1 subtype model of the famous abalone mollusc dataset (Warwick Nash, Tracy Sellers, Simon Talbot, Andrew Cawthorn, Wes Ford 1994), showing abalone body measurements as the molluscs grow. Left: Abalone measurement feature polynomials, post-fitting. Right: Loss curves during an abbreviated 250 iteration training process. Note that L1 loss is the last to be minimized, meaning that while training and validation losses become more level around step 75, the model is still performing loss arbitration between L1 and log

likelihood. This means that the polynomials are changing despite the total loss remaining level.

To fit a multiple subtype model, multiple progressions are generated as described above. During fitting, the likelihood of each record is evaluated across all stages of all subtypes, and the highest likelihood subtype and stage combination is recorded as that observation's subtype and stage. That record's nominal likelihood is the likelihood of the observation at that subtype and stage. The model's likelihood is again the cumulative product of all nominal likelihoods.

Core Assumptions

There are three assumptions which must be met to apply PPSI. Firstly, the progression of the features must be representable with polynomials. Progressive phenomena which suddenly and drastically change in a discontinuous way are not good targets for modeling with PPSI. Secondly, the features must contain enough information to distinguish where records should be placed. If there is no change in the progression, overwhelming noise in the data, or a repeating pattern such as a sine wave without any monotonic features, the model cannot distinguish where a record should be placed. Finally, all features should have an origin at zero such that the phenomenon start time corresponds to feature values of zero. As previous algorithms have done (Young et al. 2018), we achieve this using Z-scoring, where we rescale feature values to standard deviations of error from a normal control model, allowing zero to correspond to the mean value of controls.

Supplementary Material 2: Alzheimer's Study Extended Methods

Dataset Curation

Tabular multi-visit demographic/cognitive diagnosis and cerebrospinal fluid (CSF) biomarker metadata was downloaded from LONI. Rolling join was performed on participant-

specific resident ID (RID) and the dates of cognitive diagnosis and CSF biomarker acquisition for the left and right data tables respectively. Observation with absolute time difference in days between cognitive diagnosis and CSF biomarker acquisition of greater than or equal to 90 days were excluded, leaving 2907 observations across 1594 individuals. MRI and PET imaging metadata were downloaded from LONI, with the MRI metadata filtered to only the rows referring to MPRAGE MRI acquisitions and PET metadata filtered into 2 separate tables, one referring to AV45 amyloid PET scans and the other referring FDG glucose PET scans. MRI, AV45, and FDG scans were joined unto diagnosis and CSF biomarker table sequentially with a rolling join, each time dropping observations for which the imaging session was 90 days or more away from the cognitive diagnosis visit. A total of 829 observations across 620 patients were retained after this filtering procedure.

Participants in this set were then split into groups, based on whether at any point abnormal cognition (MCI or DEMENTIA diagnosis) or CSF biomarker value (Hansson et al. 2018) were observed among the visits retained after our filtering procedure. Only participants with some evidence of abnormal cognition AND abnormal CSF biomarker (any of the three) during at least one of the retained visits were classified as “patients”. Only participants with no evidence of abnormal cognition AND no evidence of abnormal CSF biomarkers were classified as “controls”. In total, 307 patients and 103 controls were retained. Imaging data for all of these participants at baseline and for a subset of 72 patients at follow-up was downloaded for LONI. All data curation and patient sample selection procedures were performed using R programming language (version 4.4.1), open-source tidyverse for general purpose data manipulation, and data.tables library for bi-directional date-based rolling join functionality.

Imaging Preprocessing

For each of the downloaded MRI images, the raw DICOM or ECAT data was converted to NIFTI format and organized in a brain derived imaging data structure (BIDS) format by visit and group using open-source dcm2bids software. MRI images were automatically aligned to the anterior-commisure posterior-commisure (ACPC) plane. SynthSeg (from the FreeSurfer software suite) was used to segment ACPC aligned MRI images, and obtain regional volume measurements in tabular format. Regional volumes were divided by the estimated total intracranial volume to obtain normalized volume values.

AV45 and FDG dynamic PET images were motion corrected using inter-frame rigid-body registration. The last 2, 4, or 6 frames (depending on specific acquisition) of each dynamic image were averaged, and the averaged image was subsequently aligned to the T1w MRI structural image using rigid-body registration and resampled to MRI resolution. Cerebellar white matter labels obtained from SynthSeg were eroded using 2mm radius sphere to obtain the reference region mask for both PET images. Averaged PET images aligned to the structural MRI image were divided by the mean of voxels which fell inside the reference region mask to obtain standardized uptake value ratio (SUVR) parametric images. SynthSeg labels were subsequently used to extract mean regional SUVR values from the resulting parametric images into a tabular format. Quality control outputs were generated at each step of the imaging preprocessing procedure and examined to ensure data integrity, and diagnose issues in the imaging data. All imaging pre-processing steps were implemented as in-house Python scripts, based on open-source ANTsPyX, NiBabel, and NiLearn modules.

PPSI Modeling

A 20-fold cross-validated subtype sweep was performed for a 20-stage progression model, examining up to 3rd degree polynomial order, and up to 5 total subtypes. Model evaluation metrics were first examined across maximal allowed polynomial degree, to determine the most parsimonious polynomial complexity. Subtype sweep plots of correlation comparison, Euclidean R2, and AIC metrics over maximal polynomial degree revealed that little improvement was obtained for capturing the subtype-specific covariance matrices, fitting the multidimensional polynomial lines to the data, and economically explaining the variance in the data beyond the 2nd degree, so maximal degree of 2 was selected for further analysis. Full 6-metric subtype sweep plots for 2nd polynomial degree models revealed that substantial improvement on all metrics was obtained when going from 1 to 2 subtypes. Going beyond 2 subtypes yielded increasingly marginal improvements in silhouette score, AIC, Euclidean R2, and evaluation distance, no additional improvements were obtained for correlation comparison, and stage distribution appeared to become less uniform (Extended Data 1).

Exploratory analyses of 1, 2, and 3 subtype models suggested that qualitatively distinct and interpretable subtypes emerge with a 2-subtype model, whereas a 3-subtype model appears to mostly partition the variance in one of the 2-subtype model subtypes. To further support our choice of a 2-subtype model, we computed the 95% confidence interval on the out-of-sample (held out baseline validation set of 55) AIC (OOS-AIC) obtained for each model trained in the 1-subtype, 2-subtype, and 3-subtype cross-validation. The 1-subtype models had a mean OOS-AIC of 10364.135 (CI95=[10334.472, 10393.798]), 2-subtype models had a mean OOS-AIC of 9833.263 (CI95=[9768.513, 9898.012]), and a 3-subtype model had a mean OOS-AIC of 9945.287 (CI95=[9865.704, 10024.87]). Given that the OOS-AIC decreased from 1 to 2 subtypes but

actually increased from 2 to 3 subtype models further justifies our selection of a 2-subtype model for further analyses.

Lastly, we computed and compared the out-of-sample multidimensional variance captured in the trained-on features for the 2-subtype model relative to the variance captured in the full training set and the held-out cross-validation folds via the Euclidean R2 metric, to evaluate the generalizability of the selected model to unseen data. The 2-subtype PPSI model appeared to capture substantial multi-dimensional variance in the full training set features (R2Euc.=0.667, CI95=[0.663, 0.67]), while the captured variance in the 55 held out observations was only slightly more modest (R2Euc.=0.618, CI95=[0.615, 0.622]), which however very closely matched the point estimate obtained from held out cross-validation folds (R2Euc.=0.612, CI95=[0.574, 0.649]). In conclusion, PPSI model performance related to capturing multidimensional variance in trained-on features appears to generalize well to unseen data, and the out-of-sample performance appears to closely match that which was obtained via 20-fold cross-validation.

ADNI Longitudinal Validation

To better capture the variability of of longitudinal subtype consistency, stage monotony, and progressions between different model fits, 20 models corresponding to the folds of the 2-subtype model cross-validation were used to obtain a 95% confidence interval of the metric, and the mean of the models was tested against a null distribution. The null distribution was obtained by permuting the follow-up subtype or stage assignments, so as to disrupt the within-subject structure in the data, prior to computing the statistic - a procedure that was repeated 1000 times for each hypothesis test. The obtained null distributions were well fit by a Gaussian, so p-values were obtained by subtracting the cumulative density function of the observed mean cross-validated metric value with respect to the null distribution from 1.

Progression features used for head-to-head comparison with SuStaIn included the three CSF measures described in the main text, along with 6 a-priori defined coarse-grained regions used to average glucose and amyloid PET granular region values. Coarse-grained regions included occipital (cuneus, lateral occipital, lingual, and pericalcarine), limbic (entorhinal, isthmus cingulate, parahippocampus, hippocampus, amygdala), temporal (fusiform, inferior, middle, superior, and transversetemporal, temporal pole, insula, and banks of the superior temporal sulcus), frontal (caudal, rostral anterior cingulate, caudal, rostral middle frontal, lateral, medial orbitofrontal, paracentral, parsopercularis, parsorbitalis, and parstriangularis, precentral, superior frontal, and frontal pole), parietal (inferior, superior parietal, postcentral, precuneus, posterior cingulate, and supramarginal), and basal ganglia regions (caudate, putamen, pallidum, and accumbens area).

Clinical Validation

We performed a clustering and averaging of model weights across cross-validation folds to obtain a single representative 2-subtype PPSI model for the hierarchical logistic regression model comparison against a null hypothesis model. The null hypothesis model contained only dementia status at baseline and follow-up duration (difference in days between baseline and follow-up visits), to control for these important independent factors related to probability of dementia onset at follow-up. The alternative hypothesis model contained an additional subtype by stage interaction term, based on subtype and stage inferred at baseline with the 2-subtype PPSI model. In addition to the hierarchical model comparison, predictive performance of the null and alternative model was quantified using the area under receiver operator characteristics curve (AUROC). Lastly, the variability in predictive performance of the alternative model was obtained by fitting the logistic regression on subtype and stages obtained from individual models trained on

the different cross-validation folds of the 2-subtype 20-fold cross-validation, and obtaining the 95% confidence interval on the AUROC.

Supplementary Material 3: ADNI Clinical Validation Findings

The null hypothesis logistic regression model appeared to have an AUROC of 0.654, while the subtype * stage interaction model had AUROC of 0.854, suggesting that baseline dementia status and days follow-up duration were relatively poor predictors of dementia status at follow-up, whereas the interaction between subtype and stage features obtained at baseline are a good predictor of dementia status at follow-up. Likelihood ratio test model comparison between the null and alternative hypothesis models further supports this conclusion, since the subtype * stage interaction model appeared to offer a substantially better fit to the data than the null hypothesis model ($\chi 2=17.673$, $p<0.001$). Lastly, the mean cross-validated AUROC across the 20 models trained on the different folds of the training set was 0.824 (CI95=[0.814, 0.835]), suggesting that predictive performance of subtype and stage features derived from PPSI models is robust to slight variation in the training sample across multiple runs.

Supplementary Material 4: Breast Cancer Study

Introduction

In this study, we applied our standard recommended workflow for PPSI to a publicly available dataset and examined the results after a single iteration of the modeling cycle. We identified clinical variables in the dataset which can already be used in cancer subtyping, namely "breast_carcinoma_progesterone_receptor_status","breast_carcinoma_estrogen_receptor_status", and "lab_proc_her2_neu_immunohistochemistry_receptor_status", for use in post-hoc analysis. We then identified proteins which would directly influence these variables, namely “ERALPHA”,

“ERALPHA_pS118”, “PR”, “HER2”, and “HER2_py1248”, and held them out of training entirely. We then fit PPSI with the standard, generally applicable workflow and performed a thorough analysis of the outcome. We shall describe data collection and this modeling workflow below.

Data Collection and Filtration

The dataset was downloaded from the National Cancer Institute, Center for Cancer Data website. Protein expression and clinical data were gathered using their cohort builder tool, and records were limited to solid tissue specimens taken from breast tissue in patients diagnosed with lobular or infiltrating ductal carcinomas, and without synchronous malignancies. These patients were part of the TCGA-BRCA project.

The resulting dataset included 619 tumorous protein samples and 482 protein variables, as well as a wealth of clinical information for each record. It should be noted that the vast majority of these patients appear to be on medication or radiation therapy, which is likely to impact the results. Further filters were applied to remove patients with non-standard tissue sample codes, and to remove variables with high levels of incomplete values. This is described below.

726 patients fitting the search criteria had matching clinical data, which was gathered by extraction from downloaded patient XML documents. 619 had protein expression data, and the remainder were dropped. A further 12 were dropped when it was discovered that their protein expression data had only 254 features instead of the 482 features of the other records. 24 more records were removed because their patient barcode indicated a tissue sample originating from non-tumorous tissue. A further 8 records were dropped for having more than one sample in the dataset per participant, potentially introducing merging / clinical matching issues which were outside the scope of this analysis to resolve given the remaining data pool. This filtration left 575

protein expression records. After inner-joining with clinical data, 561 training records remained for the analysis.

Z-Scoring

The 28 non-tumorous tissue protein expression files were retained for Z-scoring. 22 matched clinical / tumorous protein entries, and were ultimately used to create the Z-scoring control model. The PPSI model was not trained on this non-tumorous tissue sample set.

Z-scoring was performed using these 22 non-tumorous samples. A separate linear model was made for each protein in the non-tumorous tissue dataset to describe its values as a function of age. This model was then applied to the tumorous tissue dataset, and the resulting values were divided by standard deviations of control dataset residuals. This scaled all tumorous protein data to be interpretable as "aged matched control standard deviations," or simply "standard deviations away from normalcy." All further discussion and analysis will reference Z-scored protein values, not raw values.

Model Fitting

In the modeling process for this dataset, the first sweep was across all mentioned variables, less those identified in the introduction of this section, creating PPSI models for 1, 2, 3, and 4 subtype models. For each subtype count, models were made with first, second, and third order polynomials. For each combination of subtype and polynomial order, 10 models are fit, totaling120 PPSI models. We then selected a subtype / sweep combination.

We selected a single order (straight lines) and three subtype model because it performed highly in correlation comparison, AIC, and model R2 while showing consistent improvement as subtypes were added. In some instances, it seemed to even outperform more complex models.

We then ran a Dropout Report on the model and dataset. Described in Supplementary Materials section 10, this report systematically tests variable importance by holding out one variable at a time, running inference without it, and recording how many records were assigned a different stage and subtype. This is performed on every feature and has been visualized in Extended Data section 3. A reasonable cutoff was selected such that variables will be retained if their absence alone, despite 475 other variables, caused a mean subtype change greater than 0.0025. The resulting variables are listed in Supplementary Material Section 6, along with their modeled outcomes.

Finally, another PPSI model sweep was performed using only those selected important variables. As before, first, second, and third order models were evaluated. However, in this second sweep, models up to six subtypes were evaluated. Ten models were made for each combination of subtype and polynomial order, totaling 180 PPSI models. These sweep outputs can be found in Extended Data section 3.

A second order, three subtype model was selected. This is because the fitting variables, AIC and R2, representing model likelihood give the dataset and distance of data from their polynomial lines respectively, gained much when increasing the polynomial order from first to second, but gained less when increasing it from second order to third. Three subtypes were selected for a more nuanced set of reasons. The fit metrics described above did not show a slope of gain that increased, which is to say that there is less advantage for increasing the subtype count beyond simply partitioning the data further. Also, in the second order sweep, there are greatly diminished returns in correlation comparison, which is how well the model lines match correlations in their subtype's raw data. Further, evaluation distance, which is how different the subtypes are from one another, is extremely consistent at three subtypes and highly inconsistent at four, indicating that some reliable fit may have been found. Of note, the lower evaluation distance score at third subtype

models may indicate that the added third subtype serves to subdivide one of the others. If we consider a four subtype model, the wide distribution of sweep metrics alludes to many different potential solutions arising. Since we seek to perform a single first iteration of our modeling process, we estimate a three subtype model is appropriate for this work. Future investigators are encouraged to delve further.

Analyses

These results were thoroughly analyzed using mostly linear models and visualized with the subject matter expert plotting functions showcased in Extended Data section 10. Aside from general exploratory data analysis, linear models were fitted to target clinical variables using stage and subtype as separate regressors.

Supplementary Material 5: PPSI Subtype Sweep Metrics

A subtype "sweep" is a collection of PPSI model fittings across a range of polynomial orders and subtype counts. As described in the main text, a default sweep covers every combination of first, second, and third order polynomials and one, two, three, and four subtype models, though this can be easily altered to expand the search. For each combination of order and subtype count, ten models are fitted by default. Since PPSI withholds a random 20% of the training data for use in validation, every model is fitted with a slightly different dataset.

We sweep across these combinations of subtypes and polynomial orders to assess the model's performance and ultimately choose the best combination to use in a final model. This is akin to "finding the elbow" in a K-means clustering, where many models are fitted with different numbers of clusters, and an evaluation metric is used to identify where additional clusters do not

yield meaningfully better clusters. In PPSI, we have developed six such metrics, each unveiling different information about the model's behavior.

We will now discuss these metrics in detail.

Stage Distribution

Stage distribution can be defined as the standard deviation of the number of assigned records binned by stage. Said another way, if we count the numbers of records in each stage (disregarding subtype), we can derive the stage distribution by taking the standard deviation of these counts. Smaller stage distribution corresponds to more evenly distributed assignments across pseudotime, and higher stage distributions correspond to uneven assignments.

The authors generally seek lower stage distributions when assessing models because erratic staging can sometimes indicate overestimation or underestimation of the number of subtypes.

Eval Distance

Eval Distance is short for "evaluation distance" and is how divergent the subtypes' polynomials are from one another in space when evaluated at every stage. At every stage, each subtype's polynomials can be evaluated to create a single point in hyperspace, and those points then have a Euclidean distance from other subtypes' evaluated points. These distances are measured pairwise, and the mean of every distance at every stage between every subtype is the model's eval distance. Critically, an additional "subtype" of points is added: a set of zeros. This permits an Eval Distance measure for single subtype models and also makes the measure fall drastically when a subtype does not deviate from zero through the stages.

In practice, this is a measure of subtype diversity. When adding an additional subtype, if the measure falls, it is either similar to another existing subtype or similar to zeros. If the measure

rises, a wholly new and different subtype has been created. Models describing a continuum of progressions may create gradually falling metrics as new subtypes are added.

Silhouette Score

Silhouette score is a common metric used in clustering endeavors, and it measures how well the data points fit into their own clusters compared to the next best cluster. In PPSI, each stage and subtype can be considered a cluster in hyperspace, so the next best cluster for each observation is that which the observation is next most likely to belong to. With this definition, we can refer mechanics-minded readers to silhouette score literature and discuss its use for PPSI below.

High silhouette scores indicate that the difference between assignment bins (stage and subtype combinations) is more extreme. When using PPSI, the authors have adopted seeking out lower silhouette scores, since we infer that the model is flowing more smoothly from one stage to the next without sudden drastic changes.

Correlation Comparison

Correlation comparison is a measure of how well the subtype's polynomials mimic the correlative relationships found in their respective assigned data. We derive it by creating a synthetic dataset using the progression, then comparing the correlation matrix of the subtype's assigned data against the correlation matrix of the synthetic model data. To create the synthetic dataset, we iteratively evaluate the subtype's polynomials across stages, creating a nominal record for each stage where the feature values are the corresponding polynomial values. We then derive the correlation matrix of this synthetic dataset and compare it to the correlation matrix of the records assigned to the subtype. This comparison is performed by flattening both matrices into vectors and taking the correlation between them. In this way, correlation comparison is actually

the Mantel Score of the subtype's assigned data and the subtype's evaluated polynomial progression. In multiple subtype models, the average correlation is taken.

When modeling with PPSI, the authors seek high correlation comparison scores, as they indicate that the subtypes' equations are reflecting the correlative relationships of their respective data more precisely.

To plot the model correlogram versus the data correlogram, see Extended Data section 7, where subject matter expert plotting functions are displayed, including "Plot Subtype Correlogram."

Model AIC

Model AIC is a measure of how likely the data is given the model, penalized by model complexity. Mathematically, AIC can be written as$AIC(model) = 2K - 2logL(model)$, where K is the number of parameters and $logL$ is log likelihood. In our case, K denotes the number of polynomial coefficients used in the model, and log likelihood of the model is the sum of all log likelihoods at each record's nominal stage and subtype (described in main text section titled "The Algorithm.") Of note, when a higher polynomial order is added, the number of parameters increases by the number of features, as each feature polynomial is granted another coefficient. Further, when an additional subtype is added, the number of parameters greatly increases as an entire new subtype's worth of coefficients are created.

Low AIC means a better fitting model. When working with PPSI, practitioners must consider when an additional subtype is describing an effect, or when additional subtypes are doing nothing more than partitioning noise.

Model R2

Another measure of goodness-of-fit, Model R2 measures what proportion of variance in the data is explained by the polynomials in the model. While each record has actual values for its features, each feature polynomial provides an estimated value at each stage and subtype. In this way, given a record's assigned stage and subtype, predictions and actuals can be derived, and R2 can be calculated.

Higher Model R2 corresponds to better fitting lines. Again, when judging a sweep of models, practitioners should find the elbow in this value. It will almost always rise as further subtypes are added, so practitioners would do well to conservatively judge when incremental gains in Model R2 do not justify adding further subtypes.

Supplementary Material 6: Cancer Study Protein Outcomes

Protein	S1 P-Value	S1 Slope	S2 P-Value	S2 Slope	S3 P-Value	S3 Slope
ACC_pS79	**0.000000**	**0.39**	**0.00315**	**0.17**	**0.00758**	**0.25**
AR	**0.000000**	**-0.57**	**0.00000**	**0.35**	0.06450	0.18
ATM_pS1981	**0.000000**	**-0.72**	**0.00000**	**-0.54**	**0.00000**	**-0.69**
BCLXL	**0.000000**	**-0.45**	**0.00483**	**0.16**	0.05390	0.19
BMK1-Erk5_pT218_Y220	0.203000	0.1	**0.00000**	**0.39**	**0.00000**	**-0.59**
BRCA2	**0.017700**	**-0.19**	0.07050	0.11	0.50400	-0.06
Calnexin	**0.000105**	**0.3**	**0.00000**	**0.29**	**0.00000**	**0.67**
CDK1	**0.014100**	**-0.19**	**0.03300**	**-0.12**	0.15500	-0.14
cGAS	**0.000005**	**0.35**	**0.00000**	**0.48**	**0.00000**	**-0.53**
CHD1L	**0.000000**	**0.7**	**0.00000**	**0.46**	0.38300	-0.08
CIITA	**0.000000**	**-0.56**	**0.00054**	**-0.2**	**0.03020**	**0.21**
CJUN_pS73	**0.001820**	**-0.24**	**0.00250**	**-0.18**	**0.00530**	**-0.27**
CLAUDIN7	0.751000	0.03	**0.00000**	**0.8**	**0.00011**	**0.36**

CMYC	**0.041600**	**-0.16**	**0.00438**	**-0.17**	**0.00000**	**-0.52**
CYCLINB1	**0.000000**	**0.59**	**0.00002**	**0.25**	**0.00000**	**0.53**
EGFR_pY1173	**0.000000**	**-0.65**	**0.00019**	**-0.22**	**0.00000**	**-0.68**
Elk1_pS383	**0.000000**	**-0.77**	**0.00000**	**-0.68**	**0.00163**	**-0.3**
Enolase-1	0.439000	0.06	**0.00060**	**-0.2**	**0.00153**	**0.3**
Enolase-2	**0.000000**	**-0.54**	0.10300	-0.1	0.05240	0.19
Erk5	**0.000516**	**0.27**	**0.00808**	**-0.15**	**0.00000**	**0.65**
EVI1	**0.028000**	**-0.17**	0.20300	-0.07	**0.00000**	**0.46**
GSK3ALPHABETA	**0.000000**	**0.67**	**0.00000**	**0.51**	**0.00000**	**0.76**
JNK_pT183Y185	**0.000000**	**-0.43**	0.14200	-0.09	**0.00073**	**-0.32**
KAP1	**0.000000**	**0.52**	**0.00000**	**0.41**	0.15100	-0.14
KU80	**0.000000**	**0.69**	**0.00000**	**0.38**	**0.00000**	**0.63**
MERIT40_pS29	**0.003870**	**-0.23**	**0.00034**	**0.21**	**0.00000**	**-0.58**
MR1	**0.000000**	**-0.54**	**0.00000**	**-0.46**	**0.00000**	**-0.88**
MRE11	**0.000000**	**-0.69**	0.10500	-0.1	**0.00000**	**-0.71**
MSH2	**0.000000**	**0.69**	**0.00000**	**0.4**	**0.00000**	**0.55**
MTSS1	0.717000	0.03	**0.00000**	**0.46**	0.27300	-0.11
MYH11	**0.000004**	**-0.35**	**0.00000**	**-0.36**	**0.00000**	**-0.59**
PKCPANBETAII_pS660	**0.000000**	**0.48**	**0.00076**	**-0.2**	**0.00000**	**0.59**
SHP2	**0.001280**	**-0.25**	**0.00005**	**-0.23**	0.46300	-0.07
SRC_pY416	0.523000	0.05	**0.01610**	**-0.14**	**0.00257**	**-0.29**
Twist	**0.027400**	**-0.17**	**0.00145**	**0.19**	**0.00000**	**-0.72**
UQCRC2	**0.000557**	**0.27**	**0.00000**	**0.53**	**0.00000**	**-0.62**
YAP_pS127	0.098500	0.13	**0.00000**	**-0.39**	0.10100	-0.16

Supplementary Material 7: Data Generator Algorithm

The data generator makes simulated progression datasets. It is a series of data structures and functions which build upon one another to form a polynomial progression that can be sampled

to produce data. We shall begin at the smallest object and work our way to a full sample-able progression structure.

In working with previous algorithms like SuStaIn, the authors observed that in naturally occurring progressions of many simultaneous variables, there appear to often be groups or clusters of variables which progress similarly, especially for variables that are strongly intercorrelated based on feature modality, spatial proximity, etc. Assuming that the shared trajectory for that set of variables can be well-described by a polynomial function, the similarity of individual variable trajectories should be reflected in the similarity of polynomial coefficients used to define the progression of each variable belonging to that cluster. These variables are represented by similar coefficients in their polynomial equations. We can replicate this clustering of variables in the ground-truth generation process by defining a set of probability distributions (corresponding to groups of strongly related variables) from which polynomial coefficients are drawn. For instance, one might sample the 2-dimensional distribution from figure S7A, to obtain a vector of quadratic polynomial coefficients: [0.5, -1.25]. This would result in the following polynomial equation being defined corresponding to the trajectory along which values for the generated variable will be sampled: $f(x)= 0.5x^2 - 1.25x$. This polynomial represents the change in some feature over the course of progression, depicted in figure S7B.

Additionally, each polynomial variable is assigned a noise distribution. This is a normal distribution centered around zero with some standard deviation assigned to the polynomial variable upon instantiation. The range of allowable noise levels can be defined during data generation. When using the polynomial to generate data, a progression time point is selected, the polynomial is evaluated at that specific point, and a noise value is drawn from the distribution to be added to

the outcome. The result is a synthetic noisy observed value of that polynomial at the selected point in the progression.

One can control the heterogeneity of features belonging to the same feature cluster by adjusting the standard deviations of the above-mentioned multidimensional distribution, which defines the variance in the shape of trajectories belonging to the same cluster. One can adjust the magnitude (or curviness) of polynomials by adjusting the means of the distributions closer or further from zero. When generating data, one defines a range of means and standard deviations which might be used in generating polynomial coefficient clusters.

If one wishes to create multiple polynomial clusters (representing different groups of strongly intercorrelated variables), they create multiple polynomial coefficient distributions, depicted in figure S7C and S7D. One can create imbalance in cluster membership of generated variables by favoring some clusters over others when sampling polynomials, which we refer to as variable cluster imbalance. Variable cluster imbalance captures potential inequality in the number of features within each group of intercorrelated variables present within a dataset, which we hypothesize will adversely affect the rate of ground-truth polynomial recovery by PPSI. is an adverse condition for PPSI. We simulate this important imbalance by allocating cluster membership on the basis of a target Atkinson inequality, since it is a single metric which can represent inequality among any number of classes.

We find that our implementation of unequal class frequency generation is not highly precise in its generations but is acceptable for random data generation. Atkinson inequality exists on a scale from 0 to 1, with 0 being perfectly even and 1 being extremely unequal (epsilon of 1). When we generate unequal classes, we generate a vector with a length equal to the number of classes and which sums to a sample size. For example, a generated inequality vector of two classes

and sample size 10 might be [9,1], or drawing class membership from [1,1,1,1,1,1,1,1,1,2], resulting in an Atkinson index of 0.4. In contrast, an inequality vector of [6,4] results in an Atkinson index of 0.02. When we evaluate the actual Atkinson inequality from our inequality generation function, we find that actual Atkinson indexes of the vectors we create are generally distributed around the target Atkinson inequality and depend upon the number of classes. We further found that setting the target Atkinson to 0.1 allows us to generate a range of inequalities which are reasonable for random data generation, especially because the number of classes may vary, altering the nature of the Atkinson calculations. In short, the inequality of our class imbalance generator is good enough for creating many synthetic progressions, but we would not recommend its use if precision is required.

Once the parameters for generating a ground truth progression is specified, and ground truth polynomial coefficients corresponding to each feature is drawn for every subtype, observations may be sampled from this generated ground-truth based on a set of additional data-generation parameters. A subtype is a collection of polynomials where each polynomial represents a single feature, or column, in the dataset of observations. This progression can be sampled by selecting a random point U(0,1) in the progression time, evaluating all polynomials' values at that point, and adding amounts drawn from each polynomial's respective noise distributions. This creates a vector of values, effectively a synthetic observation. Noise distribution reflects the variance in each progression feature which is unrelated to the progression stage and subtype. Each polynomial variable is assigned a noise distribution. This is a normal distribution centered around zero with some standard deviation assigned to the polynomial variable upon instantiation. The range of allowable noise levels can be defined during data generation. When using the polynomial to generate data, a progression time point is selected, the polynomial is evaluated at that specific

point, and a noise value is drawn from the distribution to be added to the outcome. The result is a synthetic noisy observed value of that feature at that point in the progression for a given subtype.

Another characteristic of progressive phenomena that the authors considered in the data generation process is observation sampling bias of progression time points and subtypes over time. For instance, if a disease is being studied, there may be few early patient observations because the disease is difficult to detect before symptoms become noticeable, and there may be few observations late in the disease progression due to patient mortality. Furthermore, if there is more than one subtype in the disease being studied, that sampling bias may be different between subtypes due to natural differences in prevalence of each subtype. The authors assess this sampling bias as a likely adverse condition for PPSI and simulate it by creating a distribution of sampling likelihood along the progression timeline for each independent subtype. This sampling bias is implemented as a Beta distribution with alpha and beta sampled uniformly between the sampling_bias_minmax tuple defined during generation. For this work, our generator was set to (1,4), permitting the sampling bias distributions showcased in S7E and everything in between. Programmatically, a simulated progression is a collection of one or more subtypes. When sampling from a progression, a random subtype is chosen, and a record is sampled from the subtype as described above.

The authors observe yet another adverse condition for PPSI found in naturally progressive phenomena the presence of subtype class imbalance. Returning to the disease progression example, it may often be the case that one disease subtype is more common than the other. We simulate this class imbalance again using the Atkinson class membership inequality generator previously described, again set to a target Atkinson value of 0.1.

In review, the steps to assemble a simulated progression are as follows. We generate clusters of polynomial coefficients, representing groups of strongly intercorrelated progression features. We sample them to get polynomial coefficients which define the ground truth polynomial trajectory of each progression feature. We collect polynomials together into a subtype. We collect one or more subtypes into a progression. We can then generate a record observed from the progression by 1) sampling a subtype according to their subtype class imbalances, 2) sampling a progression time point according to a beta distribution which represents stage sampling bias for that subtype, 3) evaluating the feature polynomials corresponding to the sampled subtype and timepoint, 4) sampling the noise distribution for each feature and adding it to the evaluated polynomial and adding noise, 5) recording the values into a vector, along with the ground truth selected subtype and stage, and 6) repeating the process to produce a dataset of as many synthetic observations as is desired. For each generated record, the selected subtype and progression point (pseudotime value) are appended to the record, effectively adding columns for that ground truth information in the generated dataset depicted in Figure S7F.

A fully simulated dataset can be made using either the “generate_random_experiment” function, or the “generate_walked_experiment” function, both found in the ppsi data generator script. These can be called to sample data (i.e. ‘my_experiment(100)’ to generate 100 records) or visualized using the “display_experiment” function (i.e. ‘display_experiment(my_experiment)’ to clearly display the progression.

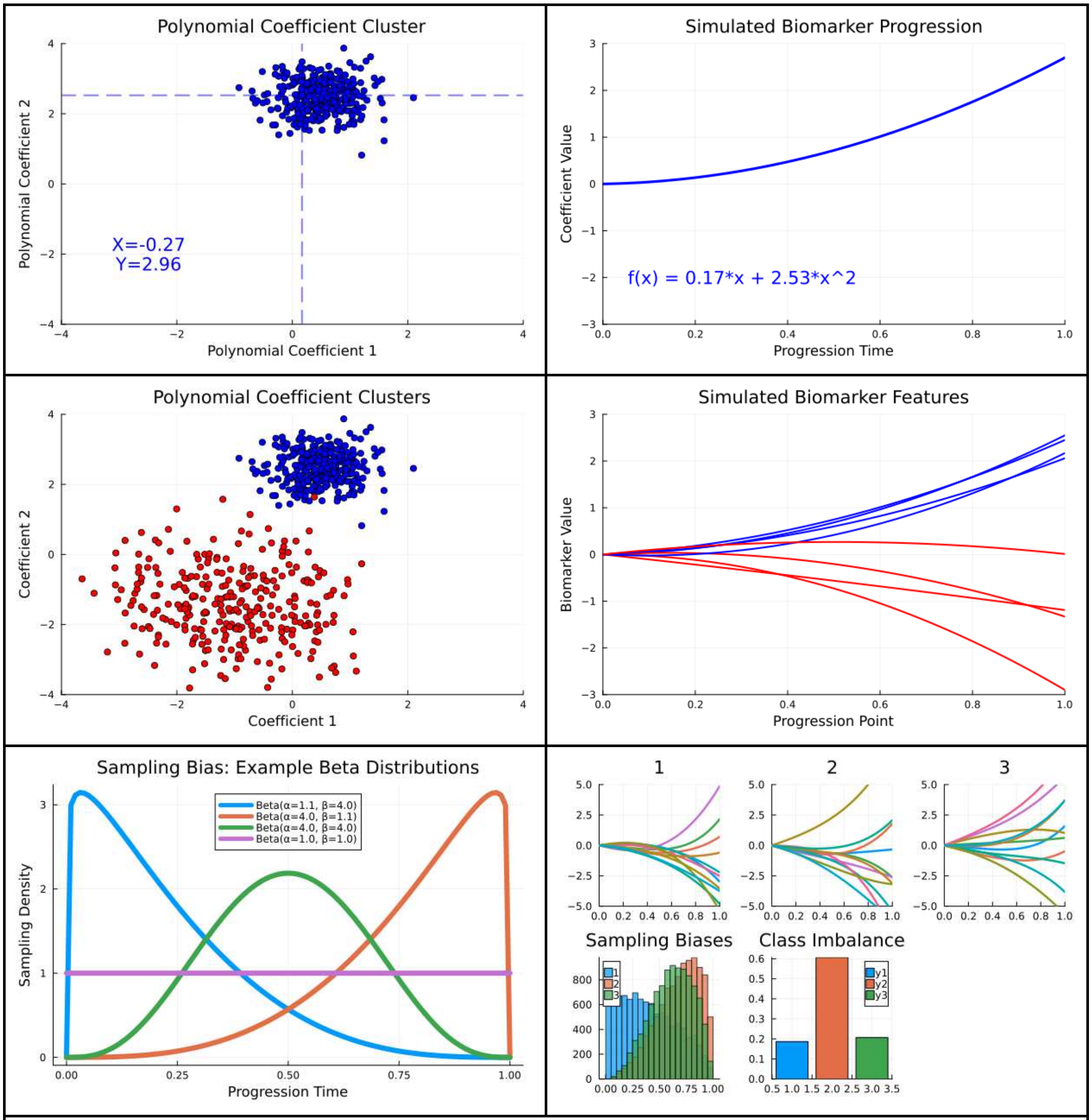

<u>Figure S7. Top Left, A:</u> A single coefficient cluster. Polynomial coefficients are drawn from these to create features. <u>Top Right, B:</u> A single simulated biomarker feature, drawn from the cluster to its left. <u>Center Left, C:</u> Two polynomial coefficient clusters, one more dispersed from the other. Groups of similar features may be drawn from this. <u>Center Right, D:</u> Multiple polynomial features generated by drawing coefficients from the distributions beside. <u>Bottom Left, E:</u> Example beta distributions, used in generating sampling bias over time. These are generated separately for each subtype. <u>Bottom Right, F:</u> A report of a generated subtype. It shows the progressions, distribution of simulated stages, and distribution of subtype assignments.

Supplementary Material 8: Simulation Study 1

Simulation Study 1 explored how dataset characteristics and model settings dictate PPSI's success in staging and subtyping records. This was done by generating 100 datasets at random and then fitting them a grid of many combinations of model settings, totaling 2,187 models per dataset. These models, and their predicted stage and subtype assignments, were collected and analyzed to assess how well the ground truth progressions could be recaptured.

Extended Methods

First, 100 datasets were generated using the data generator described in Supplementary Materials section 7, with inputs randomly drawn from the parameter spaces shown in Extended Data 6. This produced a wide range of possible progressions across a diverse field of permissive or adverse conditions. For each dataset, the selected parameters were saved for analysis, along with extracted information about each subtype, polynomial cluster, multidimensional distribution, and individual polynomial.

A grid of models was then fitted to every dataset. This grid included every combination of model parameters listed in Extended Data 6. Once the model was fitted to the dataset using the default learning rate scheduler, final inference was performed on the dataset, and the model hyperparameters and inferred stage and subtype assignments were saved for use in analysis. Of note, this study did not involve train/test splitting.

To assemble a dataset of outcomes, these tests were systematically collected and their results were concatenated into an encompassing simulation study dataset. For each of the 100 datasets, and subsequently for each of the 2,187 tests, the final predicted stages and subtypes were joined for analysis. Using this, the Adjusted Rand Index was calculated comparing the predicted versus actual subtyping, and the Pearson correlation was calculated between the predicted versus

actual staging. Furthermore, since the staging and subtyping were drawn from distributions, the actual subtype sampling bias Atkinson inequality and mean sampled stage were calculated. Simulator parameters and model hyperparameters were also saved, resulting in rows of data where each row is one test performed on one dataset, and columns were information about the test and its outcomes. These were concatenated to a dataset of 218,700 records, which were then analyzed.

Two analyses of note were performed. The first used a random forest model to extract the importance of simulation parameters and model hyperparameters in predicting the success of the model. The second applied an ordinary least squares regression to predict subtyping and staging success using one variable at a time, showing the interaction.

The random forest was applied using Pythons' Sci-Kit Learn package, with n_estimators=100, max_depth=none, min_samples_split=20, min_samples_leaf=5, max_features=1.0, criterion="squared_error", and bootstrap=true. It was fitted with all the variables listed in Extended Data 6. The feature imporances of the models were then saved for display.

Ordinary Least Squares (OLS) regression models were used to derive interactions between the above-mentioned variables and their outcomes for staging and subtyping. Python's StatsModels package was employed. For each variable, staging correlation and subtyping ari were target variables (Y) and the variable was the regressor (x). The formula for this regression was simply "Y ~ 1 + x". The summary statistics for this model were recorded.

It is important to note that when analyzing subtyping ARI, records were dropped if either their actual subtype count or their modeled subtype count was 1. This is because, in this scenario, ARI would always be 1.0.

Extended Results

Of note, the distribution of staging correlation score remained relatively level in underestimation of subtypes but sharply fell during overestimation (see Extended Data 7). Furthermore, the staging correlation score varied more when underestimated, and less when overestimated, and a Tukey HSD test (n=218,700, df=4) was used to identify differences in staging success caused by over- and underestimation of subtype count. These results are tabulated in Extended Data 8.

Measurement of subtyping success impact yielded similar results. Models with only one ground truth or modeled subtype were dropped, as the ARI of their predicted versus actual subtypes is either zero or not a number. Tukey HSD (n=99,144, df=2) of subtype discrepancy across the remaining three subtype discrepancy groups showed significant differences in ARI between all. Practically, over- and underestimation were equally detrimental, with underestimation causing a difference of -0.12 ARI, and overestimation causing a difference of -0.115 ARI.

Polynomial order discrepancy was defined as the polynomial order of the applied model minus the polynomial order of the ground truth progression, such that overestimation yields a positive number. Both values ranged from 2 to 4, so polynomial order discrepancy again ranged from -2 to 2.

Supplementary Material 9: Simulation Study 2

The second simulation study aimed to precisely extract the impact of adverse conditions on subtyping and staging success. This was achieved by holding all arguments of data generation constant except for one, scaling that argument across a range of values while quantifying modeling success.

Extended Methods

The default arguments of the data generator were as follows:

```
order = 2
mean_minmax = (-3.0, 3.0)
stdev_minmax = (1.0, 1.0)
noise_minmax = (0.5, 0.5)
n_polynomials = 20
n_clusters = 3
sampling_bias_minmax = (1.0, 1.0)
polydist_imbalance_index = 0.0
subtype_class_imbalance_index = 0.0
sample_size = 400
```

The number of subtypes was generally rotated between 1, 2, and 3 as the tests were performed across ranges of other values. This means that, if we take the sample size as an example, with its range of values from 40 to 1000 in steps of 5, there were approximately 64 tests per subtype count, evenly distributed across the range of sample sizes.

One PPSI model was fitted per generated dataset. The models were fitted with default hyperparameter values and polynomial_order = 2 (matching) and the number of subtypes equal to the correct amount for the generated dataset (also matching). Subtyping ARI and staging correlation were calculated similarly to Simulation Study 1.

Analysis was performed to determine whether there was a correlation between the changing target variable and PPSI's success metrics. Correlation tests were performed independently on staging and subtyping successes. Furthermore, these analyses were also stratified by subtype counts, ranging from 1 to 3. In other words, we determined whether there was correlation between the target variable and staging on one subtype, two subtype, and three subtype

models, and then also analyzed the impact on subtyping ARI for two and three subtype models. This totals to five correlation tests per target variable.

The list of tested variables, exact N of each analysis, correlation p-values, and correlation coefficients can be found in Extended Data section 8. These tests were all visualized and can be found in the code repository under the “notebooks_and_tutorials/ppsi_performance_plots.ipynb” notebook.

Extended Results

**Measure**	**Values**<br><br>Total Records<br>start : step size : end	**Staging Correlation**<br><br>Correlation Test, Pearson	**Subtyping ARI**<br><br>Correlation Test, Pearson
Sample Size	Total N = 193 tests<br>40 : 5 : 1000	1 Subtype: False, 0.11<br>2 Subtype: False, 0.06<br>3 Subtype: False, 0.13	2 Subtype: False, 0.14<br>3 Subtype: False, 0.13
Sampling Bias Mean Stage	Total N = 200 tests<br>N ~ 67 per subtype<br>Beta(rand(1:4), rand(1:4))<br>for each subtype	1 Subtype: False, -0.03<br>2 Subtype: False, 0.17<br>3 Subtype: False, -0.01	2 Subtype: **True**, 0.57<br>3 Subtype: **True**, 0.4
Class Imbalance	Total N = 200 tests<br>N ~ 67 per subtype<br>Random class ratios	1 Subtype: NaN, NaN<br>2 Subtype: False, 0.01<br>3 Subtype: False, 0.13	2 Subtype: **True**, 0.6<br>3 Subtype: **True**, 0.54
Feature Count	Total N = 231 tests<br>N = 77 per subtype<br>4 : 1: 80 features	1 Subtype: **True**, 0.66<br>2 Subtype: **True**, 0.62<br>3 Subtype: **True**, 0.5	2 Subtype: **True**, 0.67<br>3 Subtype: **True**, 0.6
Feature Extremity (polynomial curviness)	Total N = 197 tests<br>N ~ 66 per subtype<br>0.1 : 0.025 : 5.0	1 Subtype: **True**, 0.79<br>2 Subtype: **True**, 0.37<br>3 Subtype: **True**, 0.78	2 Subtype: **True**, 0.42<br>3 Subtype: **True**, 0.68

Feature Intra-Cluster Heterogeneity	Total N = 94 tests<br>N ~ 31 per subtype<br>0.35 : 0.05 : 5.0	1 Subtype: **True**, 0.7<br>2 Subtype: ***False****, 0.31<br>3 Subtype: **True**, 0.61	2 Subtype: **True**, 0.76<br>3 Subtype: **True**, 0.54
Noise	0.01 : 0.01 : 2.0<br>N = 187 tests	1 Subtype: ***False****, -0.02<br>2 Subtype: ***False****, -0.11<br>3 Subtype: ***False****, -0.05	2 Subtype: **True**, -0.34<br>3 Subtype: **True**, -0.35

Supplementary Material 10: PPSI Variable Dropout Report

The PPSI Julia package comes with a function intended for use in determining variable importance. The authors refer to it as a "dropout report," and the corresponding function "utilities.produce_dropout_report()" takes a trained model and a dataset as arguments. Functionally, it iteratively drops each variable from the trained model, records statistics about the model's behavior without that variable, and aggregates that information into a julia dictionary.

PPSI models, even when fully trained, can perform inference with little more than matrix math. If we drop a column from some of the matrices saved within the model struct, inference can still be performed without issue. Practically, one can train a model with all variables, then remove a variable from the model, and then still perform inference. If the predicted stages and subtypes change drastically, we can infer that the dropped variable must have been driving the answers. In this way, we can quantify variable importance.

The report is extensive and users are encouraged to analyze it within the PPSI package, though a few important concepts can be discussed here, namely stage and subtype change, R-squared change, and euclidean distance change. These are all derived from information about the original subtyping and staging, and for each variable, the modified subtyping and staging performed without that variable.

We can derive stage change and subtype change by recording the number of records whose modified stage and subtype assignment are different from the original. If 20 of 100 records changed

stage when inference was performed without a variable, then that variable has a stage change rating of 0.2. If 5 of 100 records similarly change subtype, then the variable has a subtype change rating of 0.05.

When a variable is dropped, model fit metrics can also change. The log likelihood of a staging and subtyping assignment changes, indicating which variables drive lower likelihoods. Also, if each point has some residual distance from the model, then this residual distance will change, and we can capture it by recalculating the model R2. Furthermore, one can also use this modified model to predict the value of the dropped variable, seemingly interpolating them. If all features are predicted and interpolated this way, there can be derived a euclidean distance of a model away from its interpolations.

The authors regularly use stage change and subtype change scores to determine feature importances when reducing model complexity.

<u>Supplementary Material References</u>

Tibshirani, R. 1996. “Regression Shrinkage and Selection via the Lasso.” *Journal of the Royal Statistical Society Series B-Methodological* 58 (1): 267–88.

Bottou, Léon. 2010. “Large-Scale Machine Learning with Stochastic Gradient Descent.” In *Proceedings of COMPSTAT’2010*, 177–86. Heidelberg: Physica-Verlag HD.

Nakamura, Kensuke, Bilel Derbel, Kyoung-Jae Won, and Byung-Woo Hong. 2021. “Learning-Rate Annealing Methods for Deep Neural Networks.” *Electronics* 10 (16): 2029.

Warwick Nash, Tracy Sellers, Simon Talbot, Andrew Cawthorn, Wes Ford. 1994. “Abalone.” UCI Machine Learning Repository. https://doi.org/10.24432/C55C7W.

Young, Alexandra L., Razvan V. Marinescu, Neil P. Oxtoby, Martina Bocchetta, Keir Yong, Nicholas C. Firth, David M. Cash, et al. 2018. “Uncovering the Heterogeneity and Temporal Complexity of Neurodegenerative Diseases with Subtype and Stage Inference.” *Nature Communications* 9 (1): 4273.

Hansson, Oskar, John Seibyl, Erik Stomrud, Henrik Zetterberg, John Q. Trojanowski, Tobias Bittner, Valeria Lifke, et al. 2018. “CSF Biomarkers of Alzheimer’s Disease Concord with Amyloid-β PET and Predict Clinical Progression: A Study of Fully Automated Immunoassays in BioFINDER and ADNI Cohorts.” *Alzheimer’s & Dementia: The Journal of the Alzheimer’s Association* 14 (11): 1470–81.
